# Differential Anxiety–Depression–CRP Network Structures Across Insomnia Severity Levels: Evidence From UK Biobank

**DOI:** 10.1155/da/8836588

**Published:** 2025-09-25

**Authors:** Xue Luo, Shuqiong Zheng, Yihong Cheng, Shuai Liu, Shufei Zeng, Leqin Fang, Shixu Du, Weimin Li, Hangyi Yang, Zhiting Huang, Bin Zhang

**Affiliations:** ^1^Department of Psychiatry, Sleep Medicine Center, Nanfang Hospital, Southern Medical University, Guangzhou, China; ^2^Key Laboratory of Mental Health of the Ministry of Education, Southern Medical University, Guangzhou, China

**Keywords:** anxiety, C-reactive protein, depression, insomnia, network analysis, UK biobank

## Abstract

**Background:** This study investigated the relationships between anxiety, depression symptoms, and C-reactive protein (CRP) across insomnia severity levels using network analysis and examined the structural differences within these networks.

**Methods:** Gaussian graphical model network analysis with Least Absolute Shrinkage and Selection Operator (LASSO) regularization was conducted on UK Biobank data (*N* = 143,027). Depression and anxiety symptoms were assessed using the 9-item Patient Health Questionnaire (PHQ-9) and 7-item Generalized Anxiety Disorder Scale (GAD-7), respectively. CRP was quantified using immunoturbidimetric-high-sensitivity analysis. Participants were categorized by insomnia frequency (never/rarely, sometimes, and usually). The strength symptoms and expected influence identified core symptoms, while bridge expected influence (bridge EI) determined bridge symptoms. Network comparison tests (NCTs) were performed pairwise across the three groups to assess differences in global strength and edge weights.

**Results:** Across all networks, “Depressed mood” demonstrated the highest strength centrality, while “Irritability” exhibited the highest bridge EI. “Depressed mood” had the highest expected influence centrality in the never/rarely insomnia group and “Uncontrollable worry” in other groups. NCTs revealed significant differences in global strength (*S* = 0.178, *p* < 0.01) and edge weights (*M* = 0.062, *p* < 0.01) between the never/rarely and usually insomnia groups, with stronger connections between depressive symptoms (energy/appetite) and CRP in the usually insomnia group (*p* < 0.001).

**Conclusions:** The central roles of depressed mood, uncontrollable worry, and irritability in the anxiety–depression–CRP network across all insomnia severity groups suggest that these symptoms represent potential targets for future intervention research. Notably, network structure differed across insomnia severity; the strengthened associations between depressive symptoms and CRP in the usually insomnia group suggest that insomnia severity may be an important factor to consider in understanding the relationships between affective and inflammatory processes.

## 1. Introduction

Anxiety and depressive symptoms are highly prevalent in the general population [1–4]. The World Health Organization (WHO) estimated that approximately 280 million people worldwide suffered from depression in 2019, affecting 5% of adults globally [5], while lifetime anxiety disorder prevalence reaches approximately 34% in the United States [6]. This high prevalence imposes substantial burdens on both mental and physical health. Furthermore, anxiety and depressive symptoms frequently exhibit bidirectional comorbidity with insomnia [7]; [5]. Individuals with insomnia report more severe anxiety and depressive symptoms than those without [8, 9], pointing to potential common underlying mechanisms.

Recent research implicates inflammatory markers, particularly C-reactive protein (CRP), in insomnia, anxiety, and depression. CRP, an acute-phase protein synthesized by the liver in response to inflammation, has emerged as a key biomarker for psychiatric disorders. Elevated CRP levels are consistently observed in patients with anxiety and depression [10–14]. Similarly, individuals with insomnia exhibit elevated levels of CRP, along with other pro-inflammatory cytokines (e.g., IL-6 and TNF-α) [15–17].

Importantly, insomnia severity demonstrates dose–response relationships with both psychiatric outcomes and inflammation. For instance, mild, moderate, and severe insomnia increase the risk of psychiatric diagnosis by 1.7, 2.63, and 5.04 times, respectively [18]. Kelly et al. [19] further demonstrated that insomnia severity predicts depression/anxiety severity independent of covariates (e.g., injury severity in spinal cord injury veterans). Insomnia severity also correlates positively with log-transformed CRP (log-CRP) levels [16]. While bidirectional associations between insomnia and these outcomes exist, the patterns of anxiety–depression–CRP associations within different insomnia severity groups remain unclear [19–21]. Understanding these relationship patterns is crucial for clinical practice and public health, as it could facilitate early identification of high-risk patients and enable more targeted interventions. The interconnected nature of these symptoms suggests that addressing one domain (e.g., insomnia) may yield cascading benefits across others (e.g., mood and inflammation), potentially improving both mental and physical health outcomes simultaneously.

Network analysis offers a powerful framework for this investigation. Unlike conventional approaches relying on latent variables, this method directly models relationships among observed variables, identifying core symptoms that serve as treatment targets and bridge symptoms that connect different symptom clusters [22–26]. From a mental health perspective, identifying these central drivers within symptom clusters informs precision medicine by allowing clinicians to prioritize interventions based on individual network profiles. From a physical health standpoint, mapping the anxiety–depression-inflammatory marker connections across insomnia severity levels provides vital insights into psychophysiological interplay. Critically, network analysis enables statistical comparison of structural differences, including connectivity patterns, among subgroups [27], allowing direct examination of how anxiety–depression–CRP relationships vary across insomnia severity levels [28].

The present study aims to identify core and bridge symptoms within the anxiety–depression–CRP network and to examine the structural differences in this network across groups with different insomnia severity levels in a large cohort from the UK Biobank. The findings may inform more precise identification of therapeutic targets for individuals with different insomnia severity profiles, with important implications for preventing the progression of sleep disturbances into more complex mental and physical health conditions. Understanding these network structures could guide the development of integrated treatment approaches that simultaneously address psychological symptoms and inflammatory processes, potentially improving both mental health outcomes and reducing physical health risks associated with chronic inflammation.

## 2. Methods

### 2.1. Participants and Procedure

The UK Biobank is a large population-based prospective cohort study that collected baseline data from over half a million participants, aged 40–70 years, recruited between 2006 and 2010 across 22 assessment centers in England, Scotland, and Wales [29]. At baseline, participants provided electronically signed informed consent and completed comprehensive health assessments through touch screen questionnaires, covering social, demographic, physical, and mental health indicators. Physical examinations were conducted, and biological samples were collected following standardized procedures. For the present cross-sectional analysis, we included 143,027 participants from the general population, comprising 37,362 individuals who never/rarely experienced insomnia, 68,597 who sometimes experienced insomnia, and 37,068 who usually experienced insomnia. The UK Biobank received ethical approval from the National Health Service National Research Ethics Service Northwest.

### 2.2. Measures

Depressive symptoms were evaluated using the 9-item Patient Health Questionnaire-9 (PHQ-9), which consists of nine symptoms: “Anhedonia”, “Depressed Mood,” “Sleep,” “Energy,” “Appetite,” “Guilty,” “Concentration,” “Motor,” and “Suicidal ideation.” Each item is scored on a 4-point Likert scale (0 = “not at all” to 3 = “nearly every day”), yielding a total score range of 0–27. Higher scores indicate increased severity of depressive symptoms.

To assess anxiety symptoms, we employed the 7-item Generalized Anxiety Disorder (GAD-7) scale [30], with higher scores indicating more severe anxiety. The scale comprises seven symptom domains: “Nervousness,” “Uncontrollable Worry,” “Excessive Worry,” “Trouble Relaxing,” “Restlessness,” “Irritability,” and “Negative Future Anticipation.” Each item is rated on a 4-point Likert scale (0 = “not at all” to 3 = “nearly every day”), with total scores ranging from 0 to 21. Higher scores indicate more severe anxiety symptoms.

Inflammation marker CRP levels were measured by immunoturbidimetric-high-sensitivity analysis on a Beckman Coulter AU5800. A natural logarithmic transformation was applied to the positively skewed CRP values to achieve a more symmetric distribution. CRP was treated as a continuous variable in all network analyses rather than applying clinical thresholds for categorization.

Insomnia symptoms were evaluated using a single-item questionnaire. Participants were presented with the following inquiry: “Do you have trouble falling asleep at night, or do you wake up in the middle of the night?” The response options provided were “never/rarely,” “sometimes,” “usually,” and “prefer not to answer,” allowing for the assessment of insomnia frequency.

For our analysis, we categorized participants into three groups based on their responses: never/rarely experiencing insomnia, sometimes experiencing insomnia, and usually experiencing insomnia. Prior to statistical analysis, participants with missing values on any of these measures were excluded to ensure data accuracy.

### 2.3. Statistical Analyses

#### 2.3.1. Network Estimation

Network analyses were performed using the R program (version 4.4.1), with the bootnet and qgraph packages [31]. In this approach, symptoms are depicted as nodes within the network, while the connections between them are represented as edges [32]. These nodes encompassed depressive and anxiety symptoms, as well as CRP levels, for each category, as detailed in Table S1.

The network models were regularized using the Least Absolute Shrinkage and Selection Operator (LASSO) technique to improve the interpretability of the findings and to reduce the likelihood of spurious connections [31, 33, 34]. For model selection with the Extended Bayesian Information Criterion (EBIC), the tuning parameter was set to 0.5, balancing sensitivity and specificity in edge selection [35, 36].

To assess the significance of each node within the network, we calculated centrality measures including strength, expected influence, betweenness, and closeness [37–40]. The calculations were carried out utilizing the “centralityPlot” function in the qgraph package [41]. Centrality values were normalized using z-scores to facilitate comparisons among nodes.

To identify bridge symptoms linking different symptom clusters within the network, we used the “bridge” function from the networktools package [34]. We employed bridge expected influence (bridge EI) as the primary indicator to quantify the bridge centrality of symptoms. Recognizing these bridge symptoms is instrumental in uncovering the key nodes that regulate the dissemination of information throughout the network [42, 43]. The network visualization was created by employing the Fruchterman–Reingold algorithm [44]. The predictability of the nodes within the network model was assessed using the R package “mgm” [45].

#### 2.3.2. Estimation of Network Accuracy and Stability

The robustness of the network solution was evaluated by examining the precision of edge weights and the stability of centrality indices using the “bootnet” R package. Confidence intervals (CIs) for the edge weights were determined through nonparametric bootstrapping [46, 47]. After the initial analysis, the dataset underwent random resampling to produce new datasets, from which 95% CIs were derived. The width of the CIs indicates the level of uncertainty in the estimation of the network parameters, with tighter CIs suggesting a more reliable and stable network [31].

To evaluate differences in network properties, we performed 1000 permutations and used a bootstrap differential test to assess variations in edge weights and node centrality indices [48]. Subset bootstrapping was used to evaluate the stability of the centrality indices. This process involves randomly selecting data subsets, recalculating the centrality indices multiple times, and quantifying their stability by determining correlation stability coefficients (CS-C) across the different subsets [49]. CS-C values exceeding 0.25, and preferably above 0.50, are generally recommended to indicate adequately stable centrality indices [31].

#### 2.3.3. Comparison of Networks Among Never/Rarely, Sometimes, and Usually Experiencing Insomnia Groups

Insomnia severity was categorized into three levels: never/rarely, sometimes, and usually experiencing insomnia. Differences among the networks for these three insomnia severity levels were evaluated using the network comparison test (NCT) from the NetworkComparisonTest R package [50]. The NCT uses a two-tailed permutation test to assess differences between two networks based on several invariance metrics [50, 51]. Specifically, this test was used to examine disparities between the networks in terms of: (1) Overall network strength: Calculated as the sum of the absolute values of all edge weights. (2) Network structure: Characterized by the distribution of edge weights within each network and the comparative strength of individual edges, adjusted for multiple comparisons [39]. Statistical significance was determined at *p*  < 0.05 for two-tailed tests.

## 3. Results

Table 1 presents the demographic characteristics for the general population and three groups categorized by insomnia frequency (never/rarely, sometimes, and usually experiencing insomnia). The sample included variables of sex, age, depression symptoms (PHQ-9), anxiety symptoms (GAD-7), and inflammation marker (CRP, both original and log-transformed values) across different insomnia severity groups.

### 3.1. Anxiety, Depression, and CRP Network

#### 3.1.1. Network Structures

**Figure 1:**
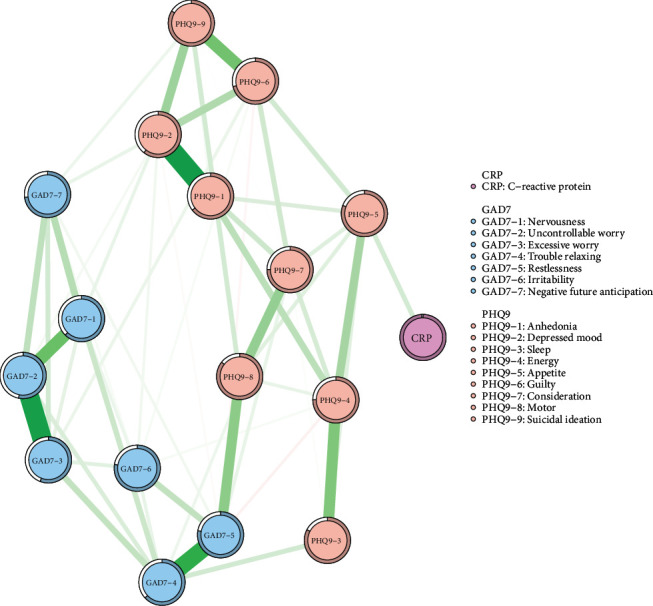
Symptom network of depression, anxiety, and inflammatory marker CRP in UK Biobank. In the diagram, symptom nodes with stronger connections are positioned closer to each other.

The analysis of the network consisting of 17 nodes, as shown in Figure 1, revealed that 112 out of 136 edges (82.35%) were estimated to be nonzero, indicating considerable interconnectedness between these symptoms. The three largest edges were between GAD7−2 and GAD7−3 (“Uncontrollable worry”–“Excessive worry”), PHQ9−1 and PHQ9−2 (“Anhedonia”–“Depressed mood”), and GAD7−1 and GAD7−2 (“Nervousness”–“Uncontrollable worry”). Edges with the highest weights were distributed within the individual communities for depression, anxiety, and CRP, as shown in Figure 1

The predictability values of the nodes ranged from 1.7% to 71.7%, with an average of 45.24%, suggesting that approximately 45.24% of the variability in nodes within the network could be explained by their neighboring nodes (Table S1). GAD7−2 (“Uncontrollable worry”) had the highest predictability, while CRP had the lowest predictability (Table S1). Notably, CRP demonstrated the lowest predictability, indicating that its variability is primarily explained by factors external to the anxiety–depression symptom network. This pattern is consistent with the characteristics of peripheral inflammatory markers, as CRP levels are influenced by multiple physiological, genetic, and environmental factors.

Figure S1 illustrates the centrality measures of all nodes. The symptom with the highest strength centrality was PHQ9−2 (“Depressed mood”), followed by GAD7−4 (“Trouble relaxing”), and GAD7−2 (“Uncontrollable worry”). Analyses of expected influence centrality revealed that PHQ9−2 (“Depressed mood”) occurred most frequently on the shortest path between other nodes, followed by GAD7−2 (“Uncontrollable worry”) and GAD7−4 (“Trouble relaxing”) (Figure S1).

For bridge symptoms, GAD7−5 (“Restlessness”), GAD7−6 (“Irritability”), and GAD7−4 (“Trouble relaxing”) had the highest bridge strength values. GAD7−6 (“Irritability”), GAD7−4 (“Trouble relaxing”), and GAD7−5 (“Restlessness”) also had high bridge EI values (Figure S2).

#### 3.1.2. Network Accuracy and Stability

Figure S3 illustrates the stability of the centrality indices using a case-dropping subset bootstrap analysis. The CS-C for strength and expected influence were both 0.75 (Figure S3).

To assess the accuracy and stability of the network estimation, a bootstrapping process with 2500 iterations was employed (Figure S4, S5). Figure S5 shows the results of the bootstrapped difference tests. The edge weights of PHQ9-1-PHQ9−2 (“Anhedonia”–“Depressed mood”) and GAD7-2-GAD7−3 (“Uncontrollable worry”–“Excessive worry”) were significantly greater than those of the other edges.

The nonparametric bootstrapped difference test for strength demonstrated that node PHQ9−2 (“Depressed mood”) had the greatest strength compared to the other nodes (Figure S6).

### 3.2. Anxiety, Depression, and CRP Network: Insights From Insomnia Severity

#### 3.2.1. Network Structures

**Figure 2:**
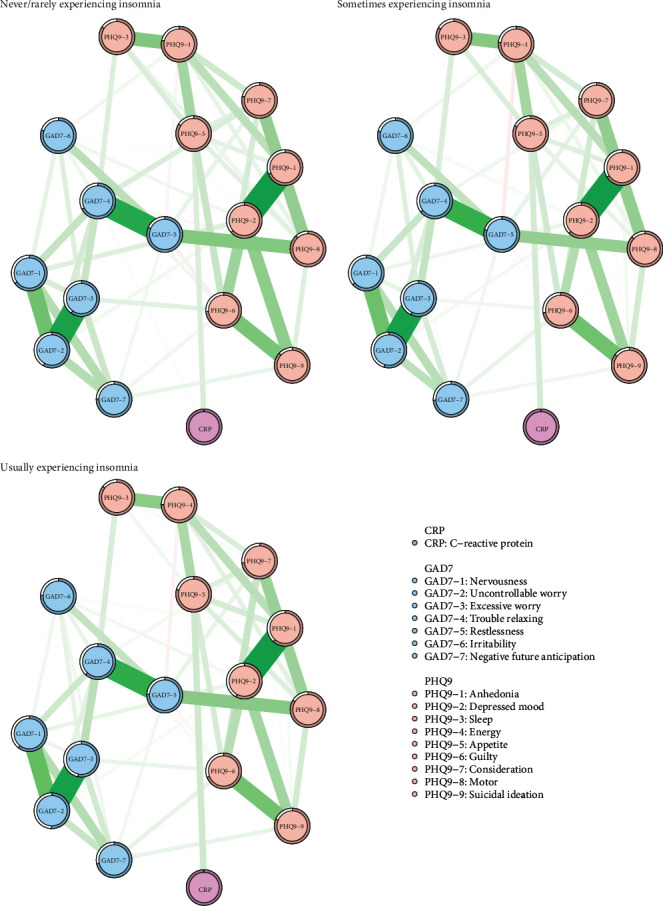
Symptom network of depression, anxiety, and CRP in UK Biobank categorized by insomnia severity.

The network analysis consisting of 17 nodes (Figure 2) revealed that 113 out of 136 edges (83.09%), 112 out of 136 edges (82.35%), and 110 out of 136 edges (80.88%) were estimated to be nonzero edges in the never/rarely, sometimes, and usually experiencing insomnia networks, respectively. This indicates considerable interconnectedness between symptoms. Node predictability varied across insomnia severity levels. In the never/rarely experiencing insomnia group, node predictability ranged from 1.1% to 66.4% (mean: 40.92%); in the sometimes experiencing insomnia group, from 1.4% to 70.3% (mean: 43.36%); and in the usually experiencing insomnia group, from 2.6% to 74.5% (mean: 47.41%). These averages indicate the proportion of node variability explicable by neighboring nodes in each network, suggesting that predictability enhances as the severity of insomnia increases (Table S2–S4).

**Figure 3:**
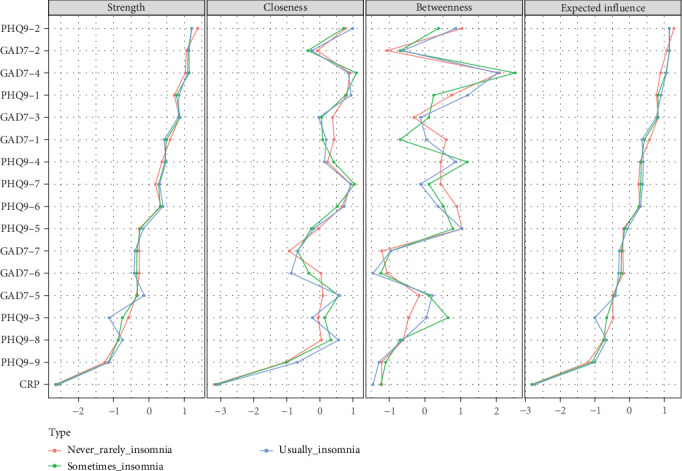
Centrality indices for the network of depression, anxiety, and CRP in UK Biobank categorized by insomnia severity.

Figure 3 illustrates the centrality measures of all nodes categorized by insomnia severity. The three strongest edges were between GAD7−2 and GAD7−3 (“Uncontrollable worry”–“Excessive worry”), PHQ9−1 and PHQ9−2 (“Anhedonia”–“Depressed mood”), and GAD7−1 and GAD7−2 (“Nervousness”–“Uncontrollable worry”) across all network.

In the never/rarely experiencing insomnia networks, PHQ9−2 (“Depressed mood”) exhibited the highest strength centrality, followed by GAD7−2 (“Uncontrollable worry”) and GAD7−4 (“Trouble relaxing”). Expected influence centrality analysis showed that the most frequent nodes on the shortest paths were PHQ9−2 (“Depressed mood”), GAD7−2 (“Uncontrollable worry”), and GAD7−4 (“Trouble relaxing”; Figure S7). For bridge symptoms, GAD7−6 (“Irritability”) had the highest bridge strength values, followed by GAD7−5 (“Restlessness”) and PHQ9−2 (“Depressed mood”). GAD7−6 (“Irritability”), PHQ9−6 (“Guilty”), and PHQ9−2 (“Depressed mood”) also had high bridge EI values (Figure S8).

In the sometimes experiencing insomnia networks, PHQ9−2 (“Depressed mood”) had the highest strength centrality, followed by GAD7−4 (“Trouble relaxing”) and GAD7−2 (“Uncontrollable worry”). GAD7−2 (“Uncontrollable worry”) had the most frequent occurrence on the shortest path between other nodes, followed by PHQ9−2 (“Depressed mood”) and GAD7−4 (“Trouble relaxing”) (Figure S9). For bridge symptoms, GAD7−6 (“Irritability”) had the highest bridge strength values, followed by GAD7−5 (“Restlessness”) and GAD7−4 (“Trouble relaxing”). GAD7−6 (“Irritability”), GAD7−4 (“Trouble relaxing”), and GAD7−5 (“Restlessness”) also had high bridge EI values (Figure S10).

In the usually experiencing insomnia networks, the symptom with the highest strength centrality was PHQ9−2 (“Depressed mood”), followed by GAD7−2 (“Uncontrollable worry”) and GAD7−4 (“Trouble relaxing”). GAD7−2 (“Uncontrollable worry”) had the most frequent occurrence on the shortest path between other nodes, followed by PHQ9−2 (“Depressed mood”) and GAD7−4 (“Trouble relaxing”; Figure S11). For bridge symptoms, GAD7−5 (“Restlessness”) had the highest bridge strength values, followed by GAD7−6 (“Irritability”) and PHQ9−6 (“Guilty”). GAD7−6 (“Irritability”), GAD7−4 (“Trouble relaxing”), and GAD7−5 (“Restlessness”) also had high bridge EI values (Figure S12).

#### 3.2.2. Network Accuracy and Stability

Figures S13–S15 illustrate the stability of the centrality indices using a case-dropping subset bootstrap analysis. The CS-C for strength and expected influence were both 0.75 in the never/rarely, sometimes, and usually experiencing insomnia networks.

To assess the accuracy and stability of the network estimation, a bootstrapping process consisting of 2500 iterations was employed (Figures S16, S19, and S22). Figures S17, S20, and S23 show the results of the bootstrapped difference tests. The edge weights of PHQ9-1-PHQ9−2 (“Anhedonia”–“Depressed mood”) and GAD7-2-GAD7−3 (“Uncontrollable worry”–“Excessive worry”) were significantly greater than those of the other edges in the never/rarely, sometimes, and usually experiencing insomnia networks. The nonparametric bootstrapped difference test for strength demonstrated that PHQ9−2 (“Depressed mood”) had the greatest strength compared to the other nodes in all three insomnia networks (Figures S18, S21, S24).

#### 3.2.3. Network Comparisons

**Figure 4:**
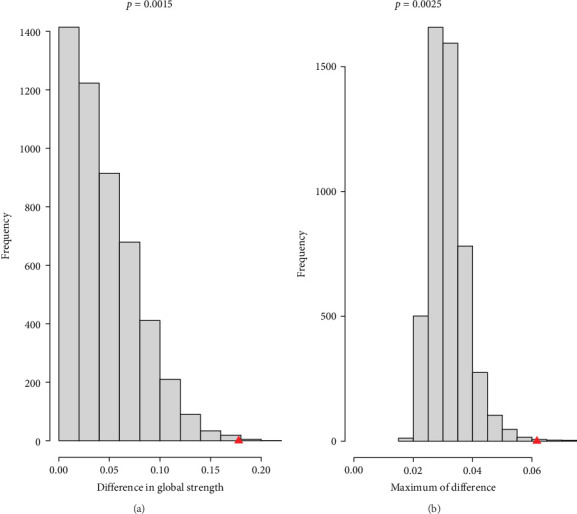
NCT results (never/rarely experiencing insomnia vs. usually experiencing insomnia). (A) Network global invariance test. (B) Network edge invariance test.

Following a comparative analysis of these three networks, no significant differences were observed in both global strength (*S* = 0.054, *p*=0.31) and edge weights (*M* = 0.032, *p*=0.28) between the networks of never/rarely experiencing insomnia and sometimes experiencing insomnia (Figure S25). The comparative analysis of sometimes and usually experiencing insomnia networks revealed no significant difference in edge weights (*M* = 0.035, *p* > 0.05), but a significant difference in the global strength (*S* = 0.123, *p* < 0.01) (Figure S26). Last, significant differences were found in both global strength (*S = 0.178*, *p* < .01) and edge weights (*M* = 0.062, *p* < .01) when comparing the networks of never/rarely experiencing insomnia and usually experiencing insomnia (Figure 4).

The Bonferroni–Holm correction was used to assess the potentially different edges between the networks of never/rarely experiencing insomnia and usually experiencing insomnia. The edges “PHQ9–3-GAD7–1” (“Sleep”–“Nervousness”), “PHQ9–4-GAD7–1” (“Energy”–“Nervousness”), “PHQ9–4-GAD7–2” (“Energy”–“Uncontrollable worry”), “PHQ9–4 and GAD7–4” (“Energy”–“Trouble relaxing”), “PHQ9–9 and GAD7–5” (Suicidal ideation–Restlessness), “PHQ9–4 and GAD7–6” (Energy–Irritability), “PHQ9–4 and CRP” (“Energy”–“immuno-metabolic markers CRP”), and “PHQ9–5-CRP” (“Appetite”–“immuno-metabolic markers CRP”) differed significantly (*p* < 0.001).

The usually experiencing insomnia didn't present edges “PHQ9–3-GAD7–1” (with a strength of 0.005 in the never/rarely experiencing insomnia network), “PHQ9–4-GAD7–1” (with a strength of 0.024 in the never/rarely experiencing insomnia network), “PHQ9–9-GAD7–5” (with a strength of 0.016 in the never/rarely experiencing insomnia network), and “PHQ9–4-GAD7–6” (with a strength of 0.02 in the never/rarely experiencing insomnia network). In contrast, the never/rarely experiencing insomnia network didn't manifest edges “PHQ9–4-GAD7–2” (with a strength of 0.004 in the usually experiencing insomnia network), “PHQ9–4-GAD7–4” (with a strength of 0.02 in the usually experiencing insomnia network), “PHQ9–4-CRP” (with a strength of 0.019 in the usually experiencing insomnia network), and “PHQ9–5-CRP” (with a strength of 0.038 in the usually experiencing insomnia network).

## 4. Discussion

This study represents the first network analysis examining structural differences in anxiety–depression–CRP networks among groups with varying degrees of insomnia severity. Key findings revealed: (1) significantly different network structures between never/rarely versus usually experiencing insomnia groups, particularly involving energy/appetite-CRP connections; (2) PHQ9−2 (“Depressed mood”) as the most central symptom across all networks; and 3) GAD7−6 (“Irritability”) as a key bridge symptom. These findings reveal that network structures of anxiety, depressive symptoms, and CRP levels differ based on insomnia severity.

Distinct differences in symptom network connections emerged between never/rarely and usually experiencing insomnia groups, particularly regarding energy levels and inflammatory markers. In the usually insomnia group, the edge weights between “PHQ9–4-GAD7–2 (Energy–Uncontrollable worry)”, “PHQ9–4-GAD7–4 (Energy–Trouble relaxing)”, “PHQ9–4-CRP (Energy–CRP)”, and “PHQ9–5-CRP (Appetite–CRP)” were significantly higher than in the group that never/rarely experienced insomnia. This strong association between “feeling tired” and “not being able to stop or control worrying” may be attributed to chronic sleep deprivation, which triggers fatigue while simultaneously compromising stress management capacity through disrupted prefrontal cortex function and heightened amygdala reactivity. This creates a vicious cycle, where persistent anxiety leads to sympathetic nervous system hyperarousal, further disrupting sleep and exacerbating fatigue [52–54].

The never/rarely experiencing insomnia group exhibited significantly higher edge weights than the usually experiencing insomnia group for the following connections: “PHQ9–3-GAD7–1” (Sleep–Nervousness), “PHQ9–4-GAD7–1” (Energy–Nervousness), “PHQ9–9-GAD7–5”(Suicidal ideation–Restlessness), and “PHQ9–4-GAD7–6” (Energy–Irritability). A plausible explanation for this result is that individuals who rarely experience insomnia tend to have stronger concerns and negative expectations about sleeplessness, which may intensify their anxiety response when encountering sometimes insomnia, thereby creating a vicious cycle of sleep disturbance.

These specific connectivity patterns reflect the neurobiological consequences of chronic sleep disruption. Persistent insomnia activates the hypothalamic–pituitary–adrenal (HPA) axis, leading to chronic cortisol elevation that simultaneously dysregulates inflammatory resolution and metabolic processes. This explains why CRP selectively connects with energy and appetite symptoms in frequent insomnia: both inflammatory signaling and metabolic regulation converge on shared brain regions, including the hypothalamus and anterior cingulate cortex, which are essential for energy homeostasis and fatigue perception [55]. The stronger energy–anxiety connections likely reflect compromised prefrontal cognitive control under chronic sleep deprivation, creating a cycle where fatigue impairs worry regulation, while persistent anxiety further disrupts sleep [56]. Supporting these findings, previous research analyzing UK Biobank and Netherlands Study of Depression and Anxiety (NESDA) data found that CRP was significantly associated with altered appetite, sleep problems, fatigue, irritability, and worry control, with increased CRP levels specifically correlated with prolonged sleep duration and increased appetite in the NESDA cohort [57].

These network connectivity differences between insomnia groups stem from chronic sleep-related dysregulation of the HPA axis and associated neuroimmune pathways [58, 59]. Beyond cortisol-mediated effects, chronic insomnia creates cascading dysfunction through disrupted neurotransmitter function, particularly serotonin, GABA, and dopamine systems, which further compromises cognitive–emotional regulation. Additionally, chronic insomnia may exacerbate emotional vulnerability, intensifying the interplay between anxiety and depression symptoms and the inflammatory marker CRP [60].

From a clinical perspective, these findings suggest that CRP may serve as a valuable biomarker for identifying individuals whose mood and physical symptoms are interconnected through inflammatory processes, particularly in the context of chronic insomnia. The sparse but selective connectivity of CRP within our networks indicates that while inflammatory markers may not be directly driven by psychological symptoms, their strategic connections with specific somatic symptoms (energy, appetite) in vulnerable populations point to potential targets for integrated interventions addressing both psychological and inflammatory components of distress. This mechanistic understanding highlights the importance of considering sleep-inflammation-mood interactions in clinical assessment and treatment planning.

Despite significant differences in edge weights across insomnia severity levels, all groups demonstrated consistent patterns in the strongest symptom connections. Key consistent connections included GAD7−2 (“Uncontrollable worry”) and GAD7-3 (“Excessive worry”), PHQ9-1 (“Anhedonia”) and PHQ9-2 (“Depressed mood”), and GAD7-1 (“Nervousness”) and GAD7-2 (“Uncontrollable worry”), revealing fundamental and stable connectivity patterns that potentially represent core pathological pathways. While insomnia severity modulates the manifestation intensity and interaction dynamics of specific symptoms (reflected in edge weight variations), it does not alter the essential symptom interconnections.

PHQ9−2 (“Depressed mood”) emerged as the symptom with the highest strength centrality across all networks, with GAD7−2 (“Uncontrollable worry”) and PHQ9−2 (“Depressed mood”) also exhibited high expected influence centrality. These findings align with prior systematic reviews identifying “Sad mood,” “Uncontrollable worry,” and “Worrying too much” as the most central symptoms, and “Sad mood,” “Restlessness,” and “Motor disturbance” as the most frequent bridge symptoms [61]. Similarly, Mullarkey et al. [48] found depressed mood highly central in adolescent depression networks, suggesting this pattern may be consistent across age groups.

The identification of these central symptoms has important therapeutic implications, as both depressed mood and uncontrollable worry are commonly targeted in cognitive behavioral therapy (CBT) approaches [62–65], indicating that existing evidence-based treatments may effectively address the most influential network nodes. These symptoms also correspond with those addressed by mindfulness-based interventions [66, 67], suggesting multiple therapeutic pathways for targeting network-central symptoms. This convergence across studies indicates that both central and bridge symptoms may represent stable therapeutic targets.

However, our identification of “Uncontrollable worry” (GAD7−2) as having high expected influence centrality differs from Contreras et al. [23], who found worry symptoms showed moderate rather than high centrality in anxiety disorder networks. This may reflect differences in sample characteristics (clinical vs. population-based) or measurement instruments. The prominence of worry symptoms in our general population sample may reflect its fundamental role in cognitive control processes, as theoretical models suggest that uncontrollable worry reflects dysfunction in prefrontal-limbic circuits essential for cognitive–emotional regulation. When these regulatory mechanisms are impaired, the inability to control worry could have cascading effects on multiple anxiety and depression symptoms.

Our analysis identified GAD7−6 (“Irritability”) as a bridge symptom connecting the anxiety–depressive–CRP network. These findings regarding bridge symptoms are consistent with some previous studies [68, 69]. However, our results are inconsistent with yet other studies [70–73]. This inconsistency may be attributed to multiple factors. Differences in sample populations could play a role, such as studies involving disabled elderly individuals [73], adults with disabilities [72], or youth survivors of childhood sexual abuse [70]. Additionally, the use of different scales to measure the same symptoms across studies may also contribute to these discrepancies [71].

The neurobiological basis for irritability's bridge function may explain its therapeutic potential. Irritability likely stems from its dependence on emotion regulation circuits, particularly orbitofrontal and ventromedial prefrontal cortex regions critical for behavioral inhibition. This symptom involves altered dopaminergic signaling and increased noradrenergic activity, creating a neurobiological profile that overlaps with both anxiety (noradrenergic hyperactivation) and depression (dopaminergic hypofunction) pathways [74–76]. This dual neurochemical involvement positions irritability as an ideal therapeutic target. Consistent with this mechanistic understanding, existing literature supports cognitive–behavioral techniques such as anger management strategies, mindfulness-based stress reduction, and emotion regulation skills training [77–79]. Furthermore, given the observed differences across insomnia severity levels, cognitive–behavioral therapy for insomnia (CBT-I) may be an important area for future investigation ([7, 20, 21]). Future research should investigate whether interventions targeting these specific symptoms can effectively reduce overall symptom severity and prevent comorbid conditions.

CRP's low predictability (1.7%) within the anxiety–depression symptom network is both theoretically expected and clinically meaningful. As a systemic inflammatory marker, CRP reflects complex physiological processes influenced by numerous factors beyond psychological symptoms, including physical health conditions, medications, genetic factors, and lifestyle variables [16, 17]. Rather than indicating a limitation, this low within-network predictability suggests that CRP functions as a bridge between psychological symptomatology and broader physiological systems, consistent with psychoneuroimmunology frameworks [57].

A notable strength of this study lies in its large sample size and examination of how the network structure of anxiety–depression symptoms and CRP levels differs across different insomnia severity levels, offering valuable insights into the underlying psychosomatic mechanisms. Nevertheless, the present investigation also has several limitations. First, although the UK Biobank represents a longitudinal cohort, the variables examined in this analysis were assessed at a single time point, precluding longitudinal examination and causal inference. Future experimental studies are warranted to establish causal relationships and evaluate targeted interventions. Second, reliance on self-reported instruments may introduce response bias, despite their established psychometric properties and clinical utility. Third, we did not exclude participants with preexisting psychiatric conditions, which may confound the interpretation of network structural differences across insomnia severity groups. Future investigations should incorporate comprehensive psychiatric histories to elucidate the independent contribution of insomnia severity to network architecture.

Our findings underscore how insomnia severity is associated with distinct patterns of connectivity among mental health symptoms and inflammatory processes, offering a novel framework for understanding the complex interrelationships among these factors. The identification of central and bridge symptoms provides valuable targets for intervention. These findings have significant implications for developing precision medicine approaches, where treatment strategies can be tailored based on individual insomnia severity profiles and central symptom networks. From a public health perspective, early insomnia detection and intervention may serve as an effective strategy for preventing the escalation of anxiety-depression-inflammation network connections.

## Figures and Tables

**Table 1:** Illustrates the baseline characteristics of the general populations categorized by different insomnia severity in the UK Biobank.

Characteristics	General population	Never/rarely experiencing insomnia	Sometimes experiencing insomnia	Usually experiencing insomnia
*N*	143,027	37,362	68,597	37,068
Sex				
Women, *N* (%)	80383 (56.20)	17011 (45.53)	40024 (58.35)	23348 (62.98)
Age				
Mean ± SD	55.87 ± 7.73	54.73 ± 8.07	55.96 ± 7.7	56.84 ± 7.28
Range	38–72	40–72	39–72	38–70
PHQ-9				
Mean ± SD	2.74 ± 3.67	1.76 ± 2.9	2.57 ± 3.39	4.06 ± 4.42
Range	0–27	0–27	0–27	0–27
GAD-7				
Mean ± SD	2.10 ± 3.35	1.41 ± 2.68	2.03 ± 3.19	2.95 ± 4.01
Range	0–21	0–21	0–21	0–21
CRP (mg/L)				
Mean ± SD	2.26 ± 3.95	2.09 ± 3.63	2.23 ± 3.94	2.46 ± 4.24
Range	0.08–78.22	0.08–74.68	0.08–78.05	0.08–78.22
CRP (logmg/L))				
Mean ± SD	0.20 ± 1.04	0.14 ± 1.03	0.18 ± 1.04	0.27 ± 1.06
Range	−2.53–4.36	−2.53–4.31	−2.53 to 4.36	−2.53 to 4.36

Abbreviations: CRP, C-reactive protein; GAD-7, generalized anxiety disorder 7-item scale; PHQ-9, patient health questionnaire-9.

## Data Availability

Data and materials are available from the corresponding author upon reasonable request.

## Supporting Information

Supporting Information This study provides Supporting Information including detailed descriptive statistics and network analyses. Tables S1–S4 present measurement item statistics (RMSE and predictability values) for the general population and three insomnia severity groups. Figures S1–S6 show comprehensive network analyses for the general population, including centrality measures, bridge centrality indices, stability analyses, and bootstrap tests. Figures S7–S12 display centrality and bridge centrality measures for each insomnia group. Figures S13–S24 provide stability analyses and bootstrap tests across all groups. Figures S25–S26 compare network properties between different insomnia severity levels.
