# Supplementary material for: Differential Anxiety–Depression–CRP Network Structures Across Insomnia Severity Levels: Evidence From UK Biobank
Source: Depress Anxiety. 2025 Sep 25;2025:8836588. doi: 10.1155/da/8836588 (PMC12490932; doi:10.1155/da/8836588)
Supplement: Supporting Information — This study provides Supporting Information including detailed descriptive statistics and network analyses. Tables S1–S4 present measurement item statistics (RMSE and predictability values) for the general population and three insomnia severity groups. Figures S1–S6 show comprehensive network analyses for the general population, including centrality measures, bridge centrality indices, stability analyses, and bootstrap tests. Figures S7–S12 display centrality and bridge centrality measures for each insomnia group. Figures S13–S24 provide stability analyses and bootstrap tests across all groups. Figures S25–S26 compare network properties between different insomnia severity levels. [file 8836588.f1.docx]

Table S1. Descriptive statistics of measurement items in general population

| Node | items | content | RMSE | Predictability (R2) |
| --- | --- | --- | --- | --- |
| PHQ9-1 | Anhedonia | Little interest or pleasure in doing things | 0.646 | 0.583 |
| PHQ9-2 | Depressed mood | Feeling down, depressed, or hopeless | 0.606 | 0.633 |
| PHQ9-3 | Sleep | Trouble falling or staying asleep, or sleeping too much | 0.827 | 0.316 |
| PHQ9-4 | Energy | Feeling tired or having little energy | 0.752 | 0.434 |
| PHQ9-5 | Appetite | Poor appetite or overeating | 0.808 | 0.346 |
| PHQ9-6 | Guilty | Feeling bad about yourself-or that you are a failure or have let yourself or your family down | 0.713 | 0.492 |
| PHQ9-7 | Concentration | Trouble concentrating on things, such as reading the newspaper or watching television | 0.752 | 0.434 |
| PHQ9-8 | Motor | Moving or speaking so slowly that other people could have noticed | 0.857 | 0.266 |
| PHQ9-9 | Suicidal ideation | Thoughts that you would be better off dead, or thoughts of hurting yourself in some way | 0.838 | 0.298 |
| GAD7-1 | Nervousness | Feeling nervous, anxious, or on edge | 0.625 | 0.609 |
| GAD7-2 | Uncontrollable worry | Not being able to stop or control worrying | 0.532 | 0.717 |
| GAD7-3 | Excessive worry | Worrying too much about different things | 0.559 | 0.688 |
| GAD7-4 | Trouble relaxing | Trouble relaxing | 0.632 | 0.601 |
| GAD7-5 | Restlessness | Being so restless that it's hard to sit still | 0.779 | 0.393 |
| GAD7-6 | Irritability | Becoming easily annoyed or irritable | 0.777 | 0.396 |
| GAD7-7 | Negative future anticipation | Feeling afraid as if something awful might happen | 0.73 | 0.467 |
| CRP | immuno-metabolic markers | C-reactive protein | 0.991 | 0.017 |

PHQ9: the 9-item Patient Health Questionnaire; GAD7: 7-item Generalized Anxiety Disorder Scale

Table S2. Descriptive statistics of measurement items in never/rarely experiencing insomnia group

| Node | items | content | RMSE | Predictability (R2) |
| --- | --- | --- | --- | --- |
| PHQ9-1 | Anhedonia | Little interest or pleasure in doing things | 0.69 | 0.524 |
| PHQ9-2 | Depressed mood | Feeling down, depressed, or hopeless | 0.633 | 0.599 |
| PHQ9-3 | Sleep | Trouble falling or staying asleep, or sleeping too much | 0.843 | 0.29 |
| PHQ9-4 | Energy | Feeling tired or having little energy | 0.784 | 0.385 |
| PHQ9-5 | Appetite | Poor appetite or overeating | 0.839 | 0.296 |
| PHQ9-6 | Guilty | Feeling bad about yourself-or that you are a failure or have let yourself or your family down | 0.738 | 0.456 |
| PHQ9-7 | Concentration | Trouble concentrating on things, such as reading the newspaper or watching television | 0.788 | 0.378 |
| PHQ9-8 | Motor | Moving or speaking so slowly that other people could have noticed | 0.882 | 0.222 |
| PHQ9-9 | Suicidal ideation | Thoughts that you would be better off dead, or thoughts of hurting yourself in some way | 0.869 | 0.246 |
| GAD7-1 | Nervousness | Feeling nervous, anxious, or on edge | 0.652 | 0.574 |
| GAD7-2 | Uncontrollable worry | Not being able to stop or control worrying | 0.58 | 0.664 |
| GAD7-3 | Excessive worry | Worrying too much about different things | 0.599 | 0.641 |
| GAD7-4 | Trouble relaxing | Trouble relaxing | 0.675 | 0.545 |
| GAD7-5 | Restlessness | Being so restless that it's hard to sit still | 0.814 | 0.338 |
| GAD7-6 | Irritability | Becoming easily annoyed or irritable | 0.802 | 0.356 |
| GAD7-7 | Negative future anticipation | Feeling afraid as if something awful might happen | 0.754 | 0.432 |
| CRP | immuno-metabolic markers | C-reactive protein | 0.994 | 0.011 |

PHQ9: the 9-item Patient Health Questionnaire; GAD7: 7-item Generalized Anxiety Disorder Scale

Table S3. Descriptive statistics of measurement items in sometimes experiencing insomnia group

| Node | items | content | RMSE | Predictability (R2) |
| --- | --- | --- | --- | --- |
| PHQ9-1 | Anhedonia | Little interest or pleasure in doing things | 0.66 | 0.565 |
| PHQ9-2 | Depressed mood | Feeling down, depressed, or hopeless | 0.622 | 0.613 |
| PHQ9-3 | Sleep | Trouble falling or staying asleep, or sleeping too much | 0.835 | 0.302 |
| PHQ9-4 | Energy | Feeling tired or having little energy | 0.767 | 0.412 |
| PHQ9-5 | Appetite | Poor appetite or overeating | 0.823 | 0.323 |
| PHQ9-6 | Guilty | Feeling bad about yourself-or that you are a failure or have let yourself or your family down | 0.729 | 0.469 |
| PHQ9-7 | Concentration | Trouble concentrating on things, such as reading the newspaper or watching television | 0.767 | 0.412 |
| PHQ9-8 | Motor | Moving or speaking so slowly that other people could have noticed | 0.87 | 0.244 |
| PHQ9-9 | Suicidal ideation | Thoughts that you would be better off dead, or thoughts of hurting yourself in some way | 0.853 | 0.273 |
| GAD7-1 | Nervousness | Feeling nervous, anxious, or on edge | 0.638 | 0.593 |
| GAD7-2 | Uncontrollable worry | Not being able to stop or control worrying | 0.545 | 0.703 |
| GAD7-3 | Excessive worry | Worrying too much about different things | 0.573 | 0.671 |
| GAD7-4 | Trouble relaxing | Trouble relaxing | 0.644 | 0.585 |
| GAD7-5 | Restlessness | Being so restless that it's hard to sit still | 0.794 | 0.369 |
| GAD7-6 | Irritability | Becoming easily annoyed or irritable | 0.79 | 0.376 |
| GAD7-7 | Negative future anticipation | Feeling afraid as if something awful might happen | 0.743 | 0.448 |
| CRP | immuno-metabolic markers | C-reactive protein | 0.993 | 0.014 |

PHQ9: the 9-item Patient Health Questionnaire; GAD7: 7-item Generalized Anxiety Disorder Scale

Table S4. Descriptive statistics of measurement items in usually experiencing insomnia

| Node | items | content | RMSE | Predictability (R2) |
| --- | --- | --- | --- | --- |
| PHQ9-1 | Anhedonia | Little interest or pleasure in doing things | 0.614 | 0.623 |
| PHQ9-2 | Depressed mood | Feeling down, depressed, or hopeless | 0.58 | 0.664 |
| PHQ9-3 | Sleep | Trouble falling or staying asleep, or sleeping too much | 0.855 | 0.268 |
| PHQ9-4 | Energy | Feeling tired or having little energy | 0.751 | 0.435 |
| PHQ9-5 | Appetite | Poor appetite or overeating | 0.792 | 0.373 |
| PHQ9-6 | Guilty | Feeling bad about yourself-or that you are a failure or have let yourself or your family down | 0.692 | 0.521 |
| PHQ9-7 | Concentration | Trouble concentrating on things, such as reading the newspaper or watching television | 0.733 | 0.463 |
| PHQ9-8 | Motor | Moving or speaking so slowly that other people could have noticed | 0.838 | 0.298 |
| PHQ9-9 | Suicidal ideation | Thoughts that you would be better off dead, or thoughts of hurting yourself in some way | 0.813 | 0.34 |
| GAD7-1 | Nervousness | Feeling nervous, anxious, or on edge | 0.605 | 0.634 |
| GAD7-2 | Uncontrollable worry | Not being able to stop or control worrying | 0.505 | 0.745 |
| GAD7-3 | Excessive worry | Worrying too much about different things | 0.534 | 0.715 |
| GAD7-4 | Trouble relaxing | Trouble relaxing | 0.619 | 0.617 |
| GAD7-5 | Restlessness | Being so restless that it's hard to sit still | 0.759 | 0.424 |
| GAD7-6 | Irritability | Becoming easily annoyed or irritable | 0.758 | 0.425 |
| GAD7-7 | Negative future anticipation | Feeling afraid as if something awful might happen | 0.715 | 0.489 |
| CRP | immuno-metabolic markers | C-reactive protein | 0.987 | 0.026 |

PHQ9: the 9-item Patient Health Questionnaire; GAD7: 7-item Generalized Anxiety Disorder Scale


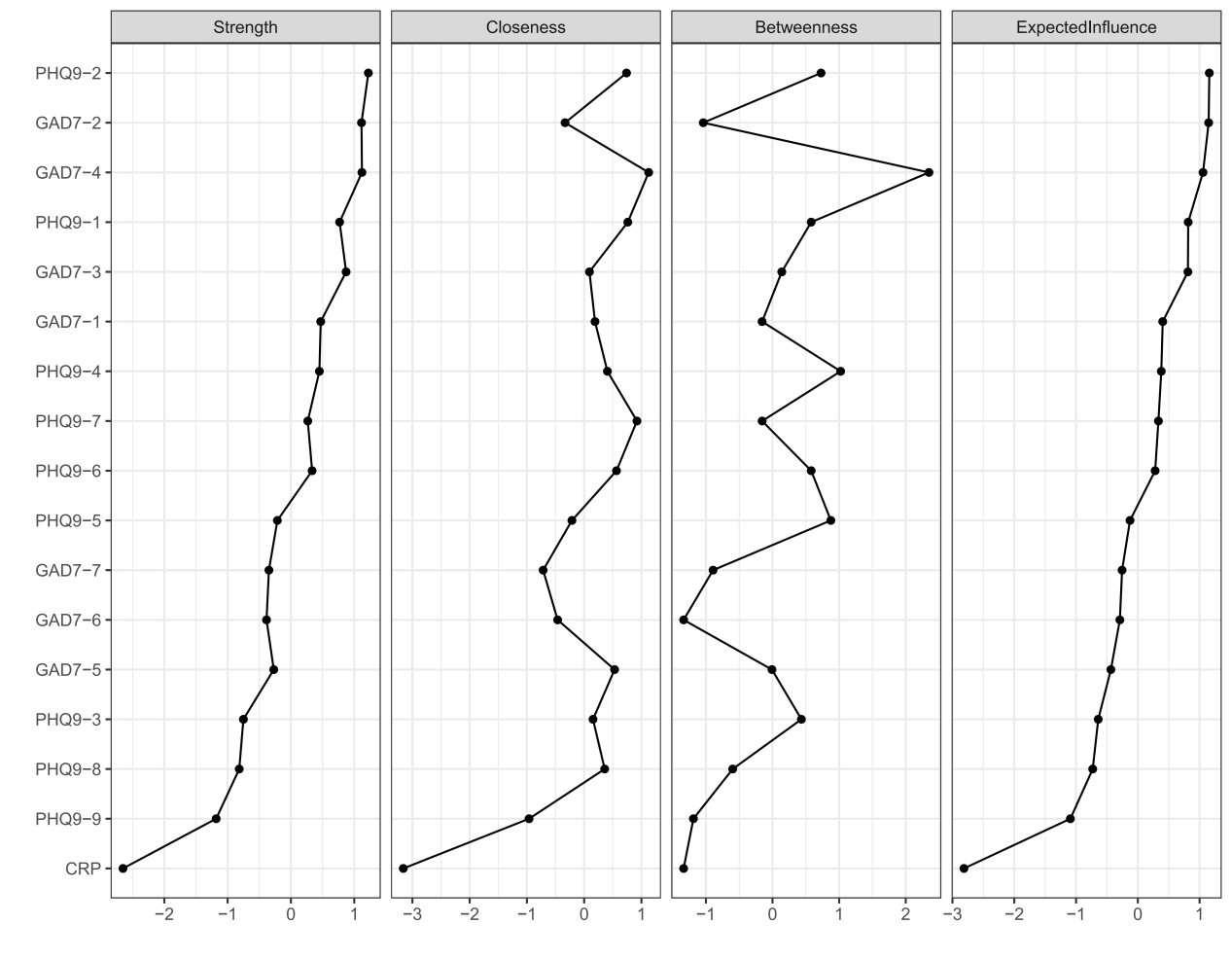


Figure S1. Centrality measures of all symptoms within the general population network.


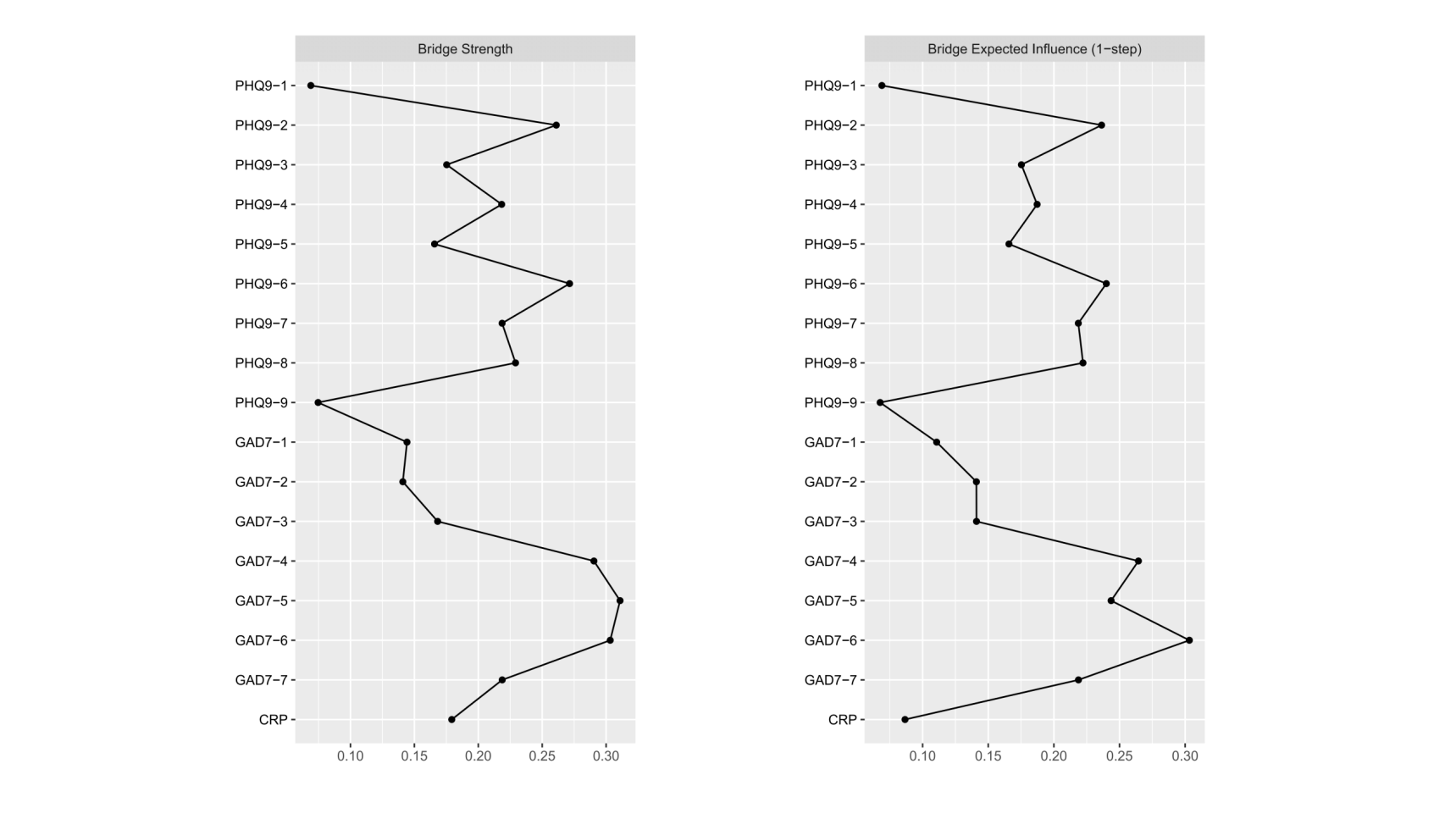


Figure S2. Bridge centrality indices for the general population network.


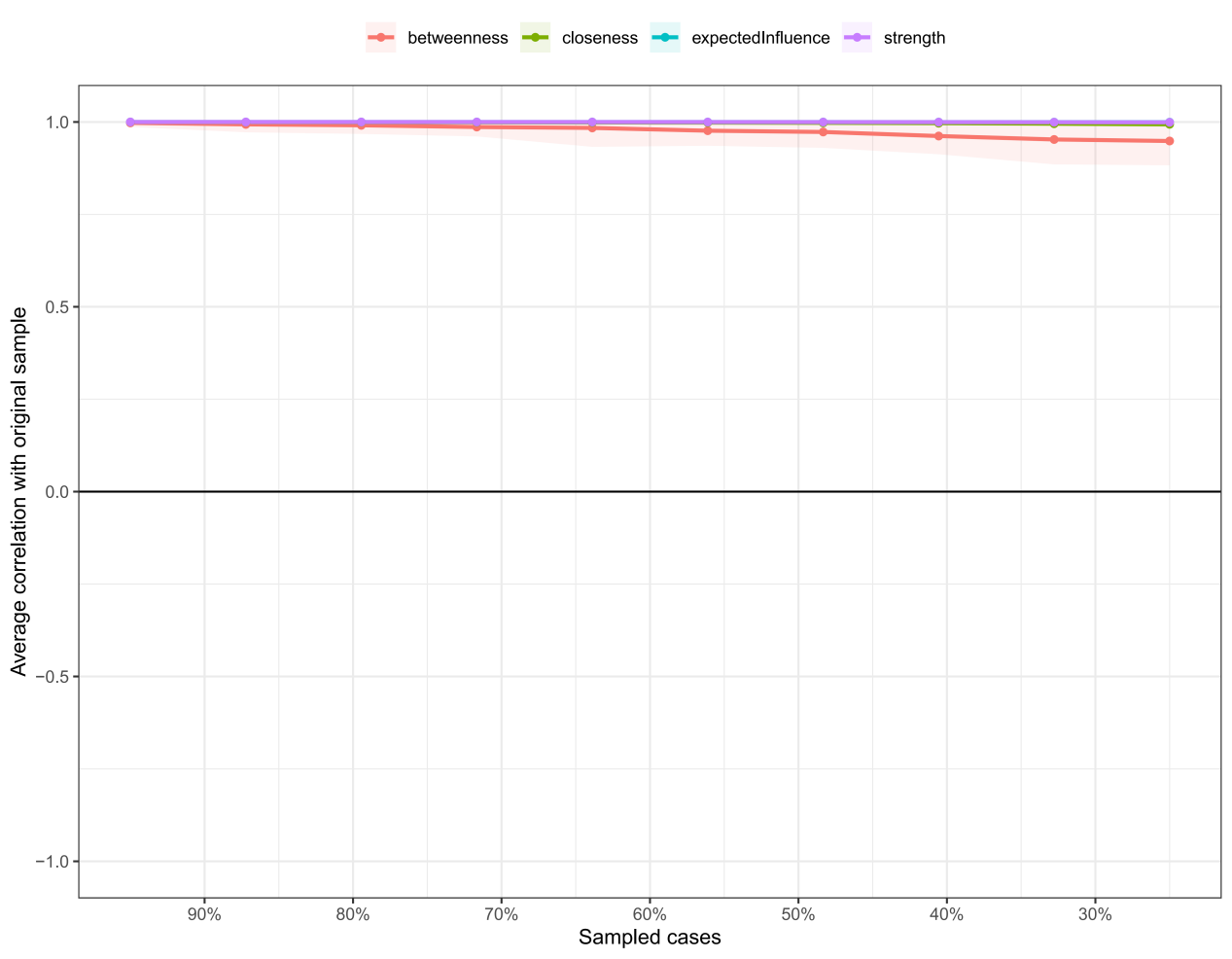


Figure S3. Stability of bridge indices by case dropping subset bootstrap in general population.


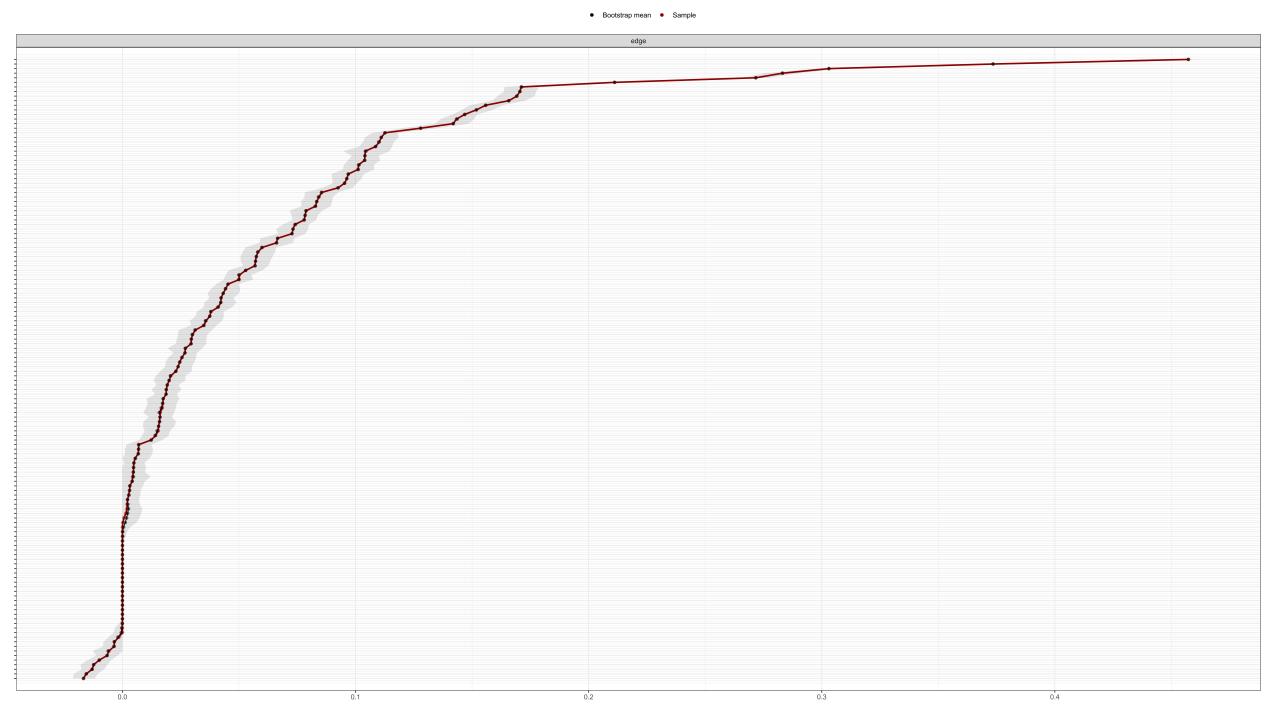


Figure S4. Bootstrapped confidence intervals of edge weights in general population.


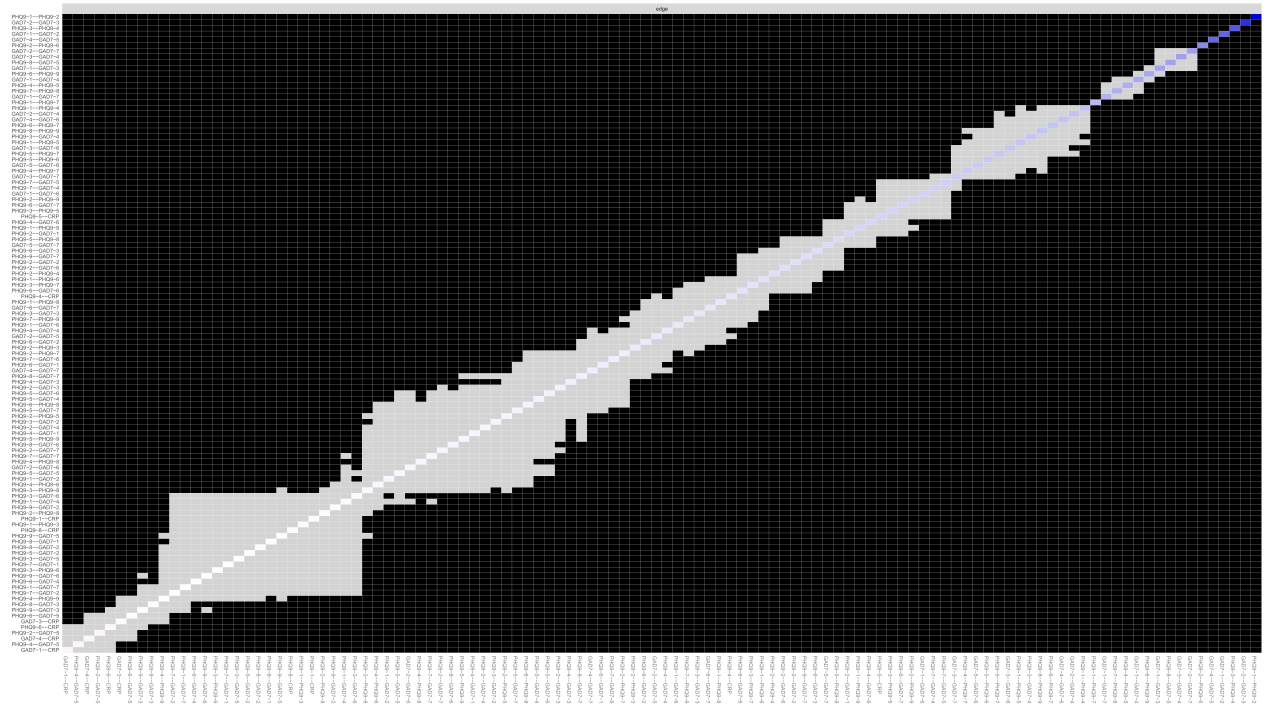


Figure S5. Estimation of edge weight difference by bootstrapped difference test in general population.


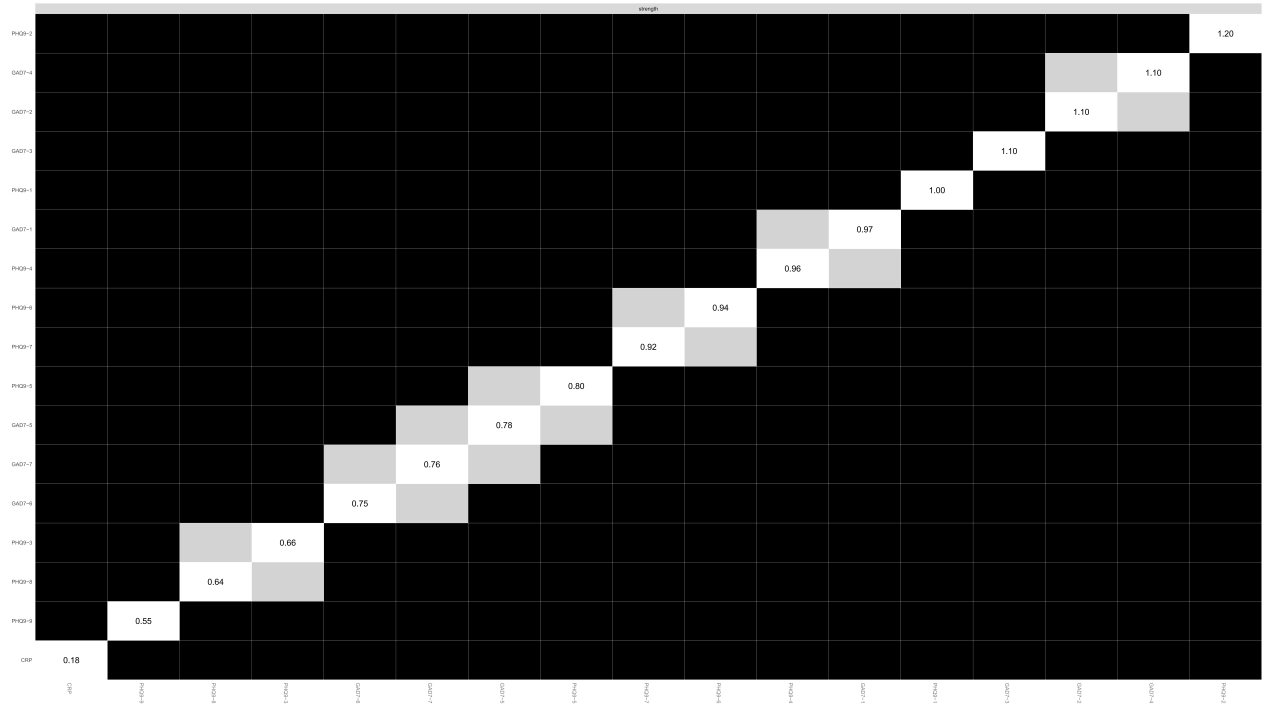


Figure S6. Estimation of node strength difference by bootstrapped difference test in general population.


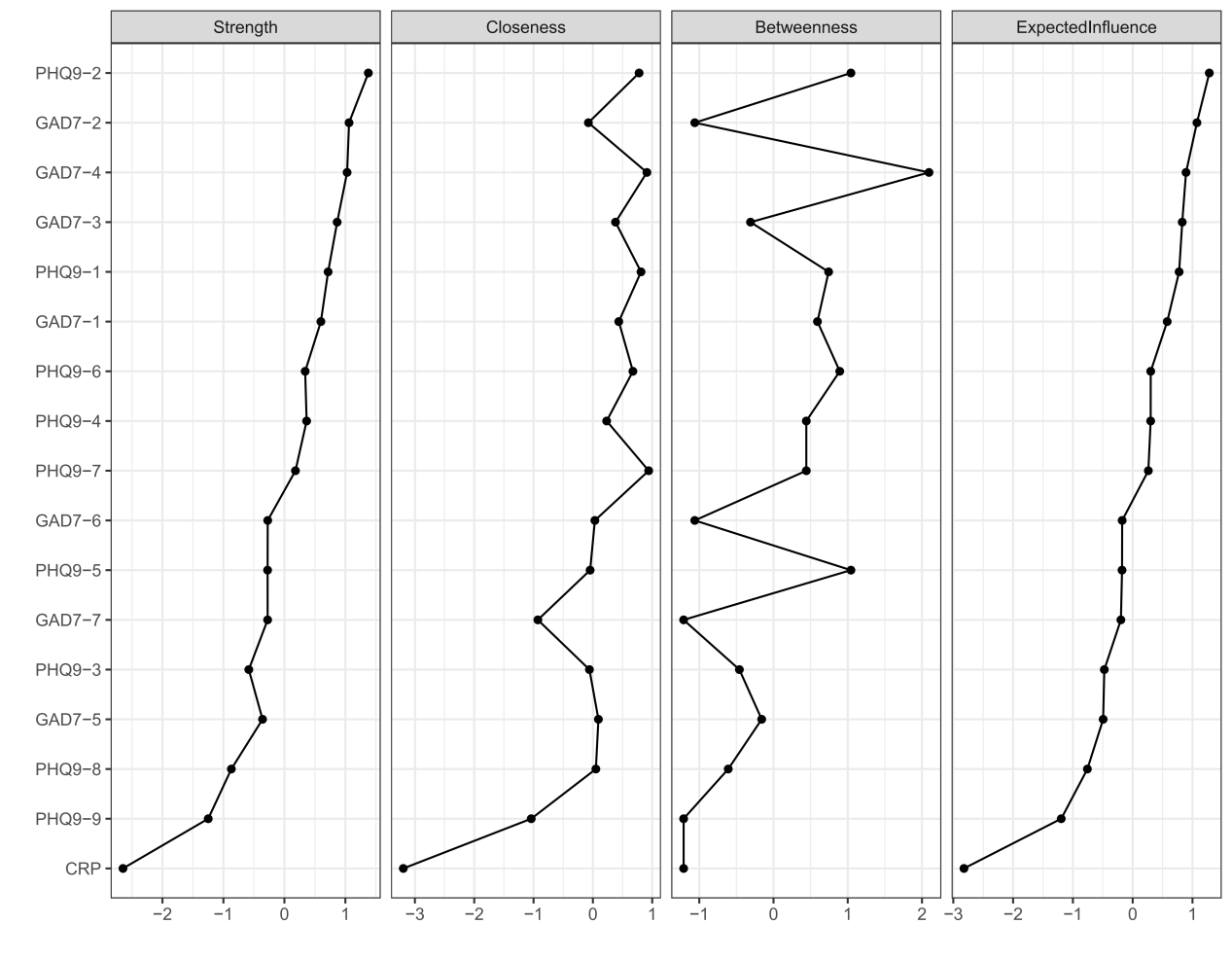


Figure S7. Centrality measures of all symptoms within the never/rarely experiencing insomnia network.


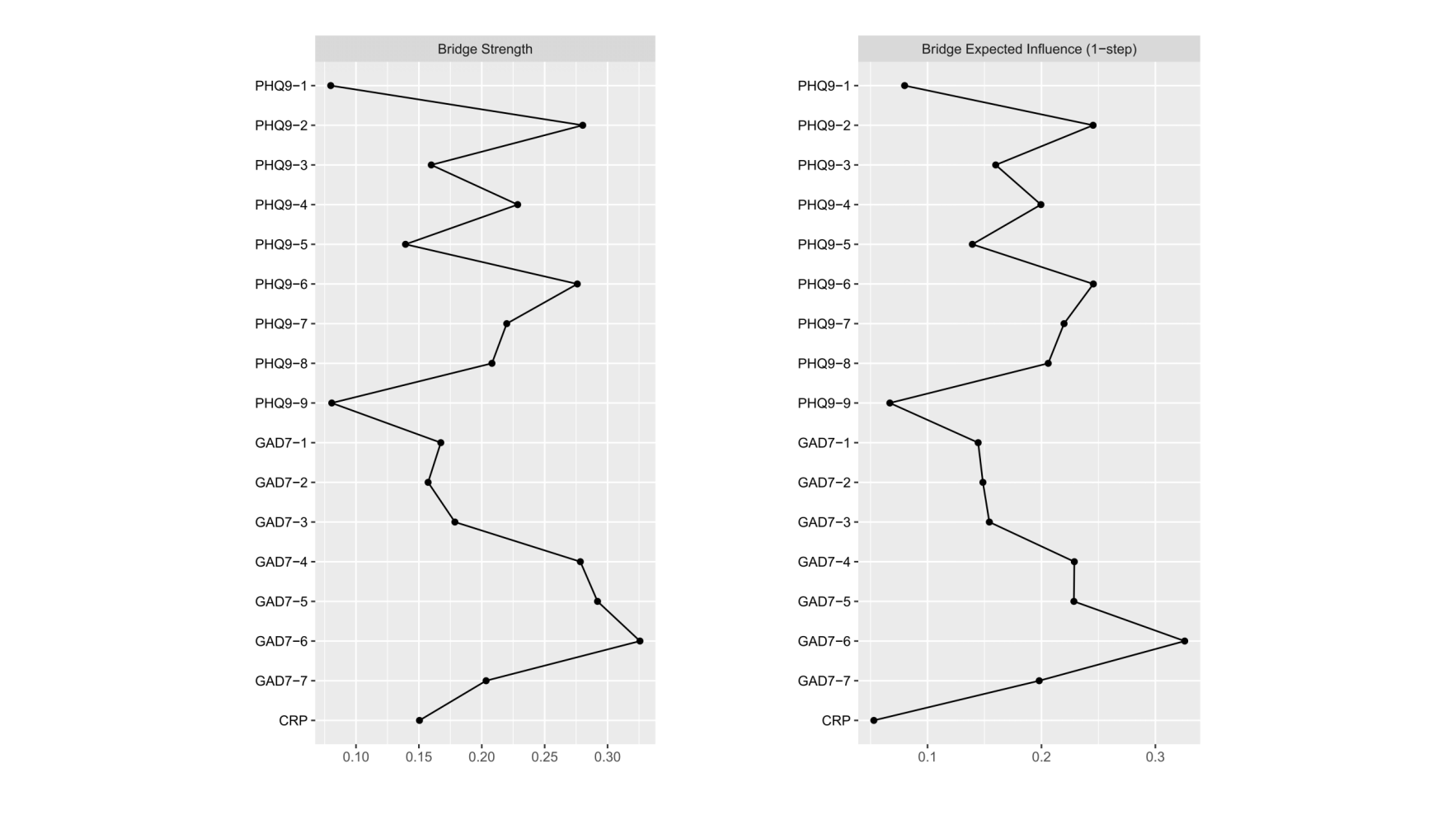


Figure S8. Bridge centrality indices for the never/rarely experiencing insomnia network.


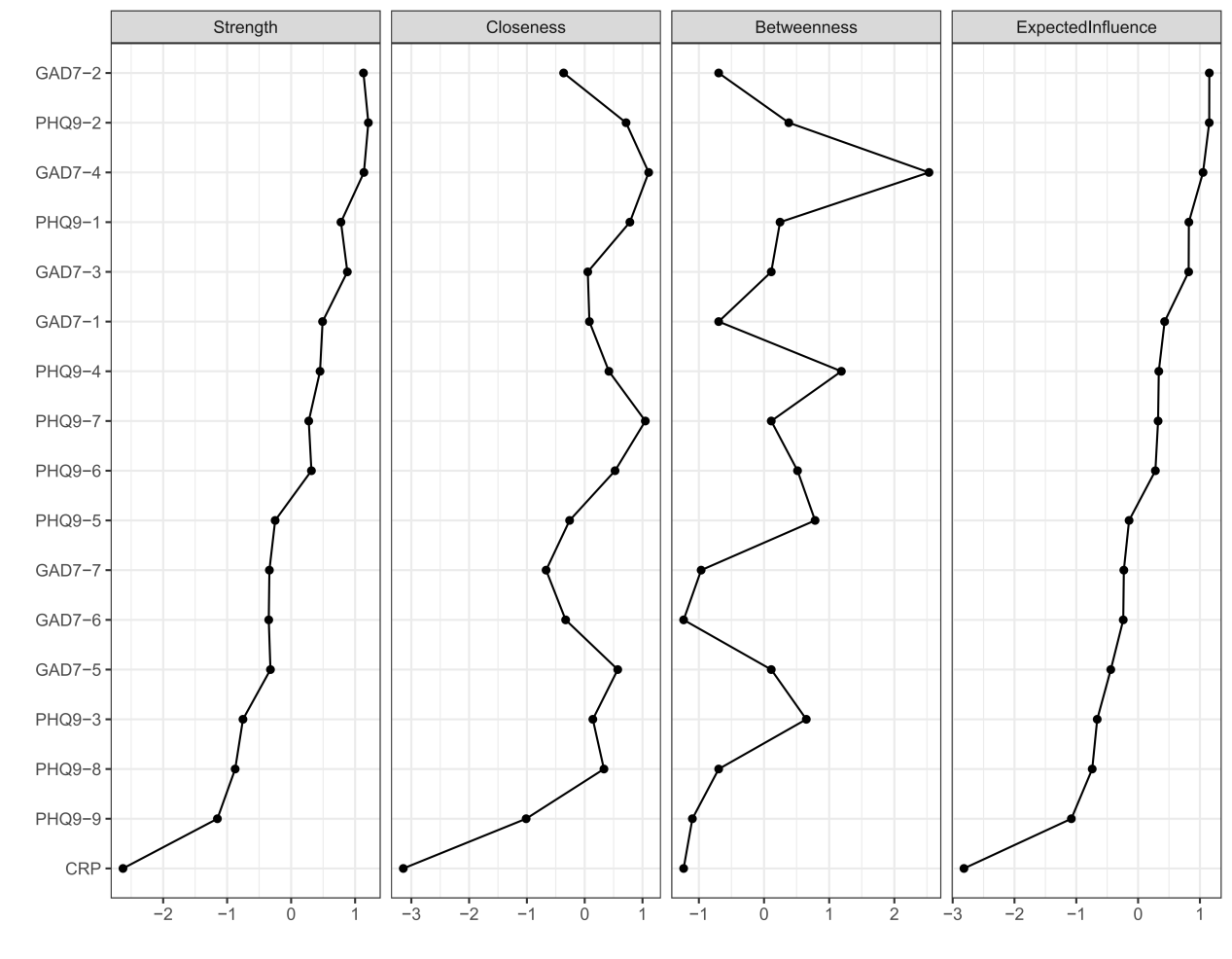


Figure S9. Centrality measures of all symptoms within the sometimes experiencing insomnia network.


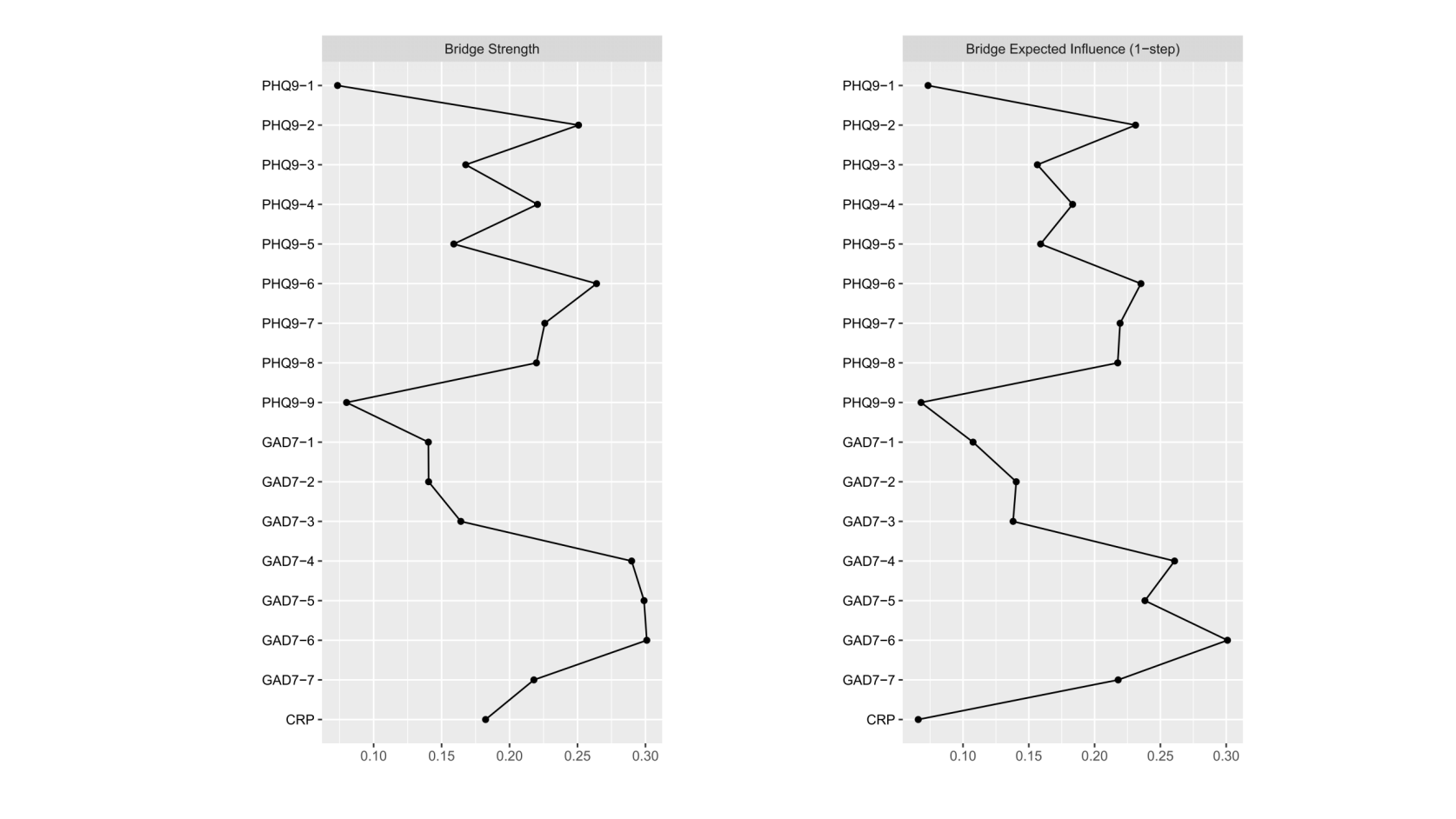


Figure S10. Bridge centrality indices for the sometimes experiencing insomnia network.


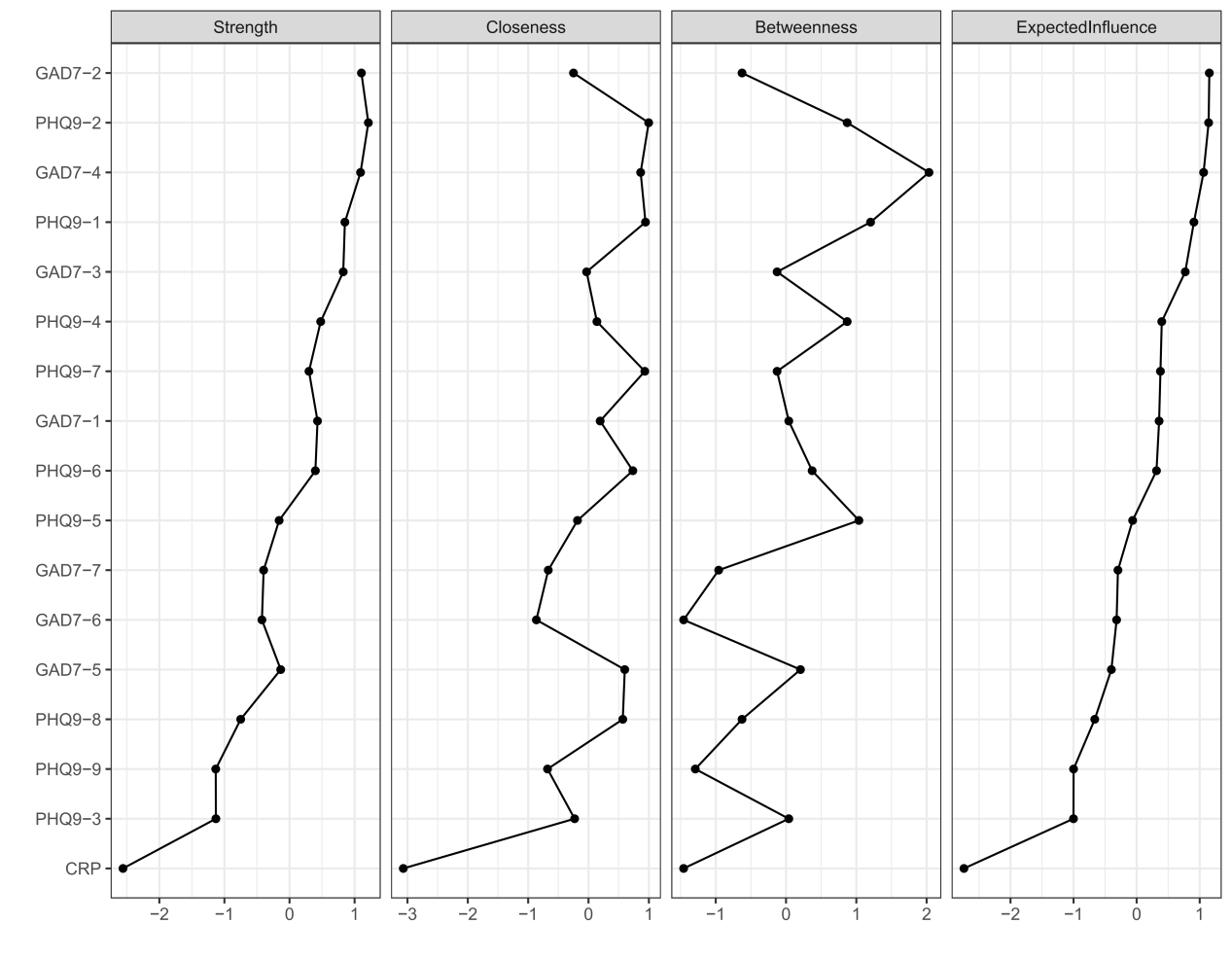


Figure S11 Centrality measures of all symptoms within the usually experiencing insomnia network.


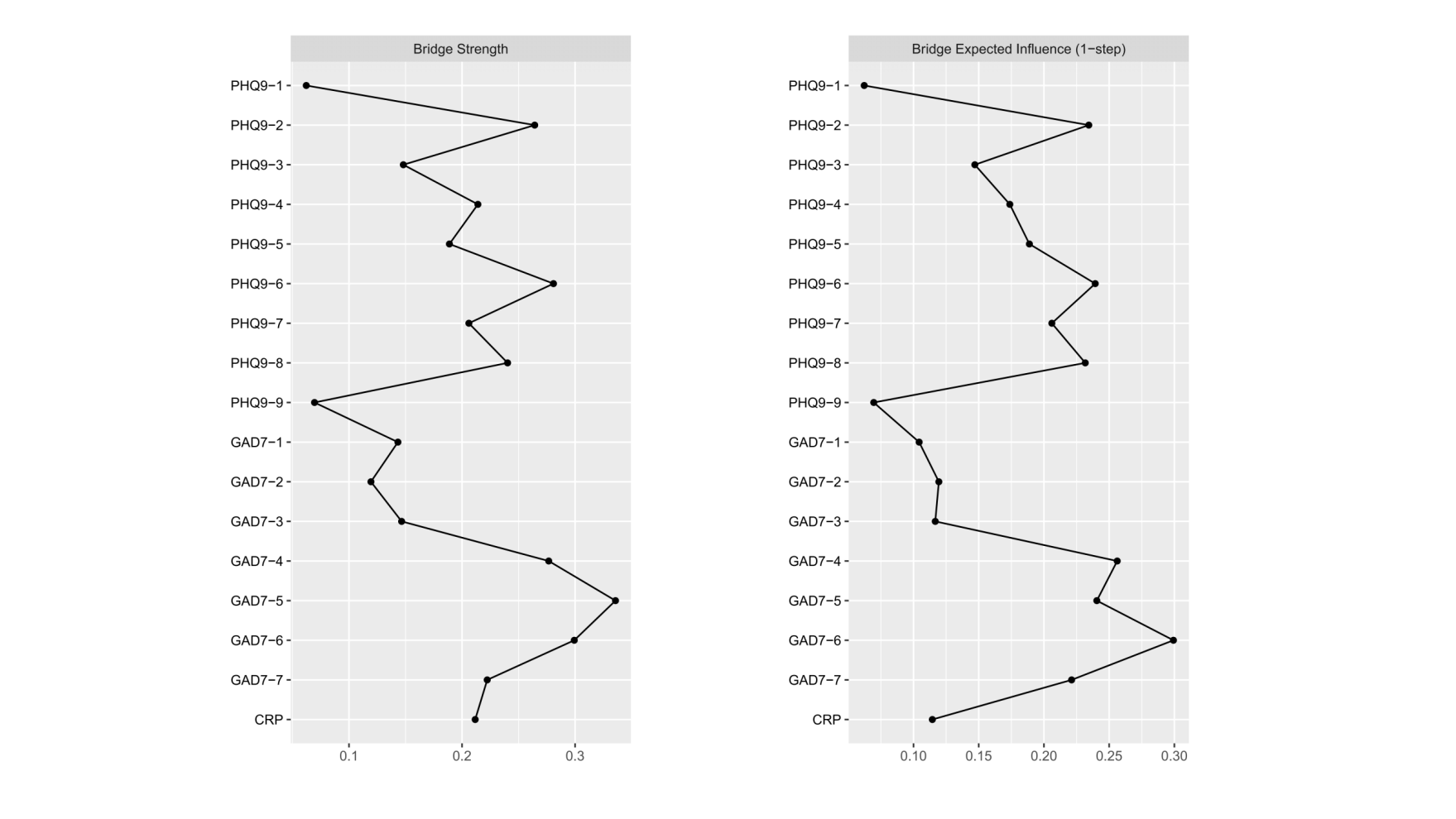
Figure S12. Bridge centrality indices for the usually experiencing insomnia network.


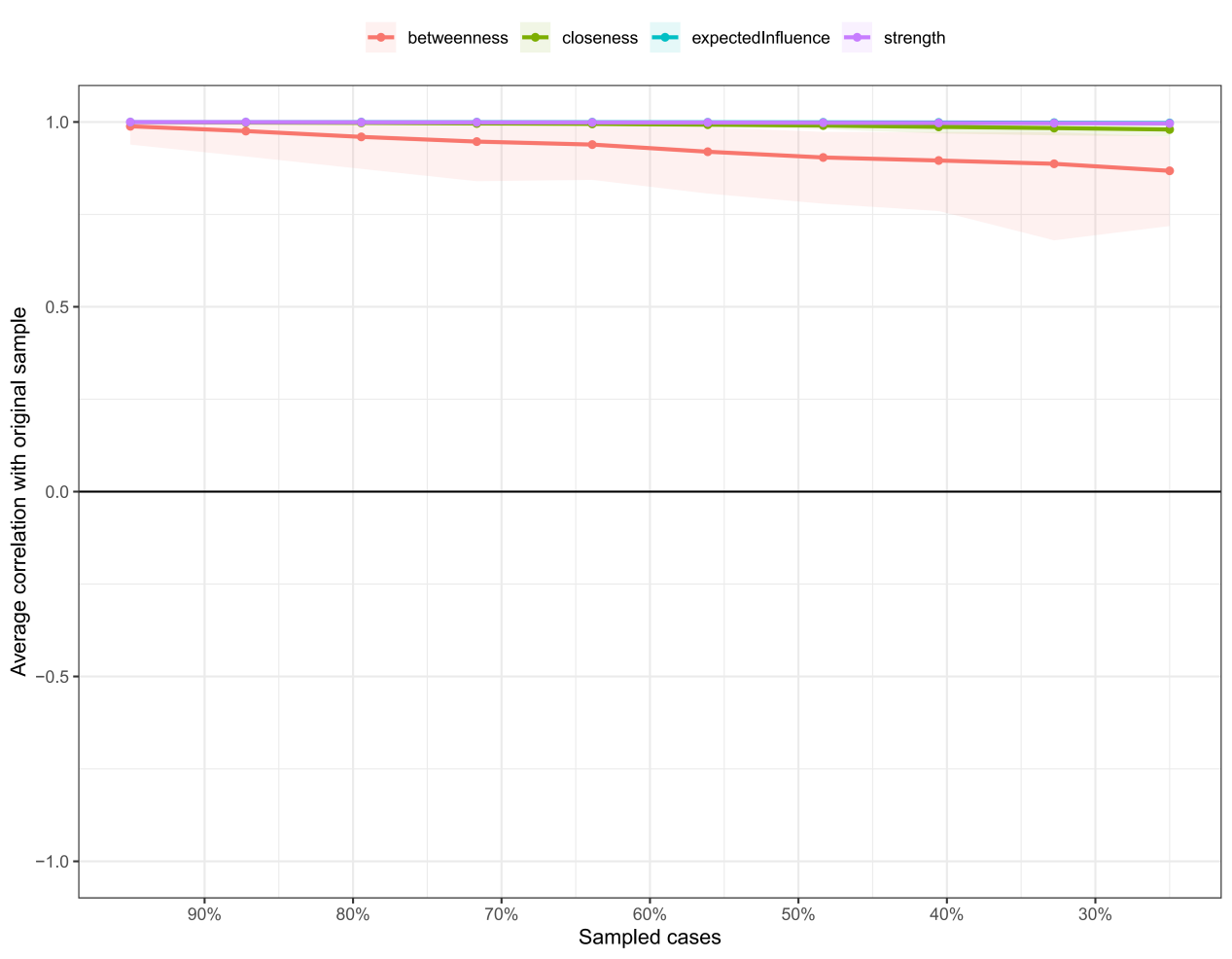


Figure S13. Stability of bridge indices by case dropping subset bootstrap in never/rarely experiencing insomnia group.


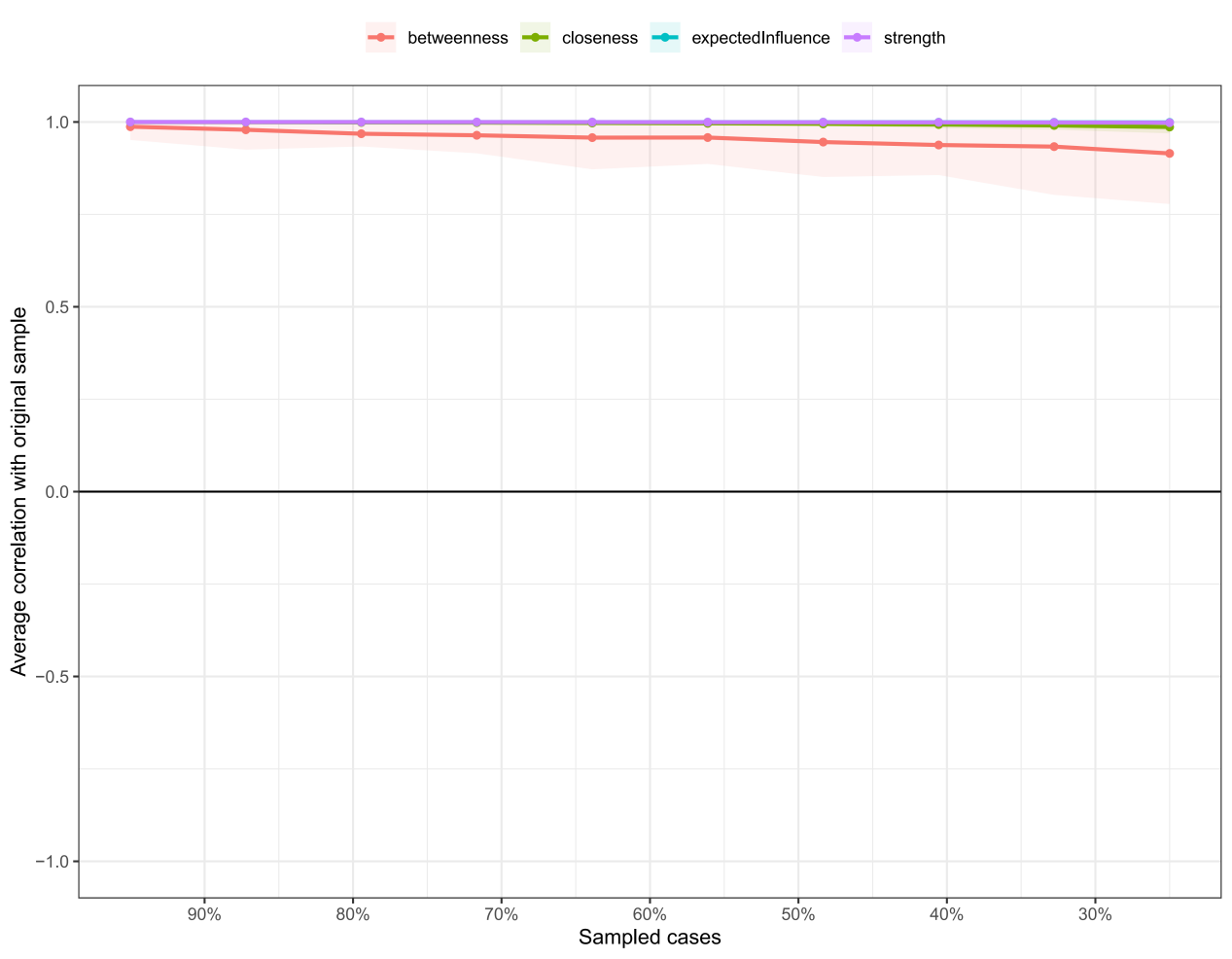


Figure S14. Stability of bridge indices by case dropping subset bootstrap in sometimes experiencing insomnia group.


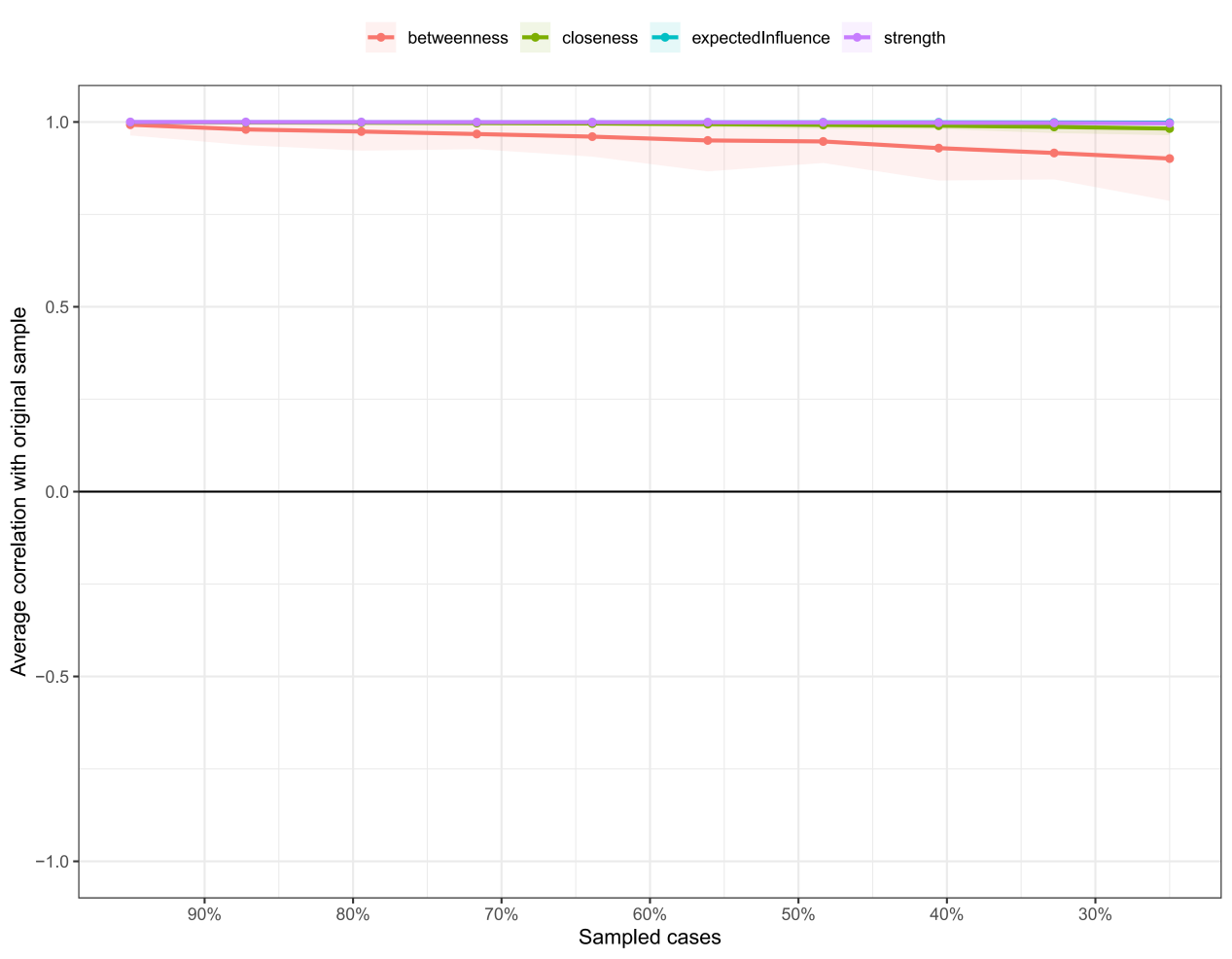


Figure S15. Stability of bridge indices by case dropping subset bootstrap in usually experiencing insomnia group.


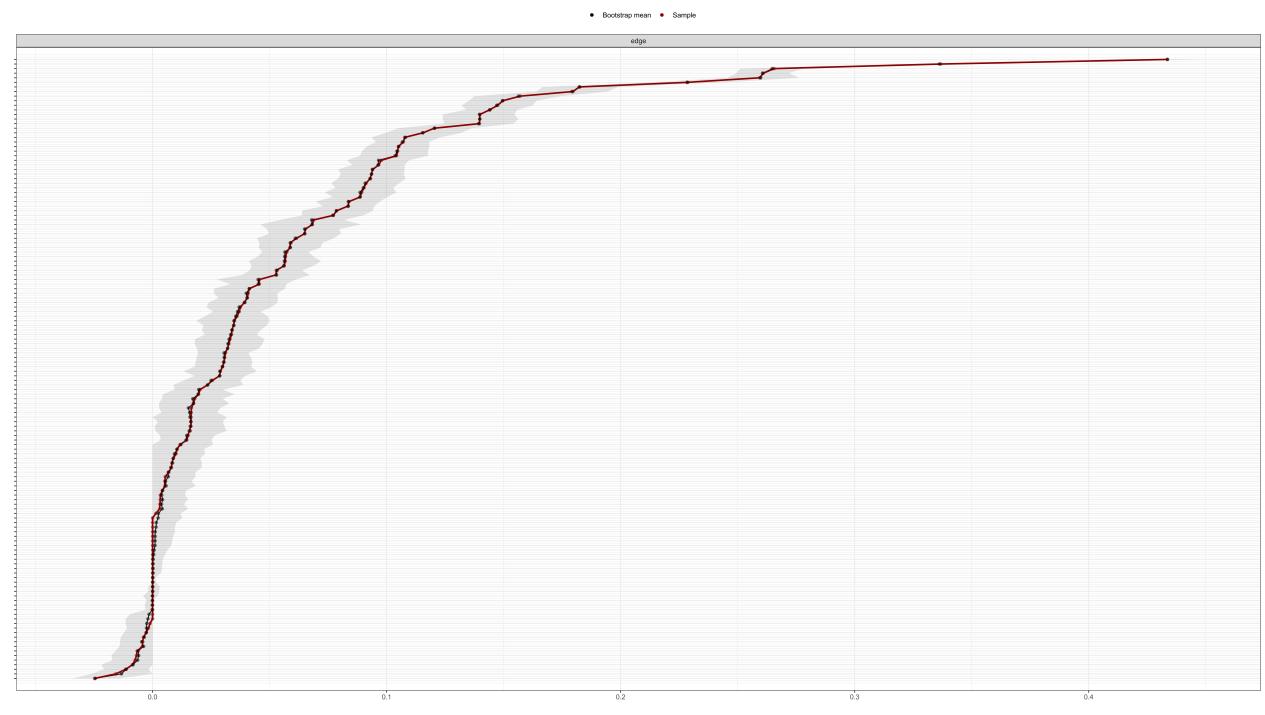


Figure S16. Bootstrapped confidence intervals of edge weights in never/rarely experiencing insomnia group.


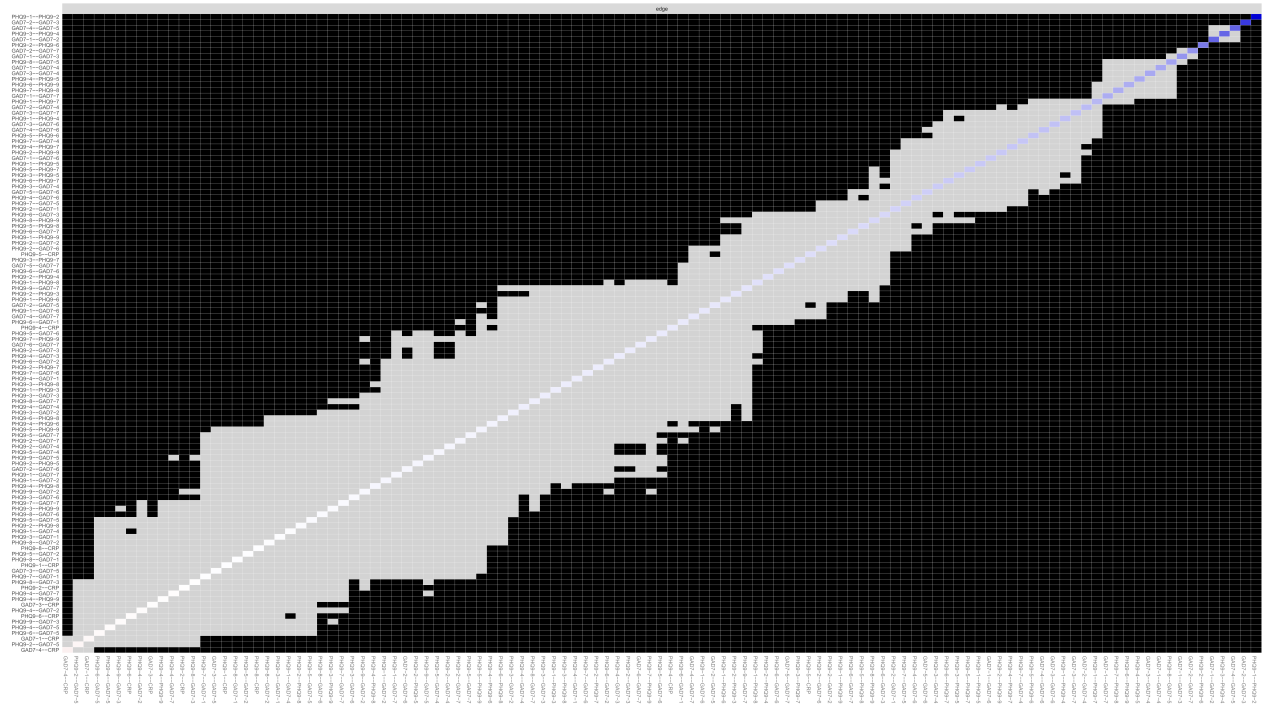


Figure S17. Estimation of edge weight difference by bootstrapped difference test in never/rarely experiencing insomnia group.


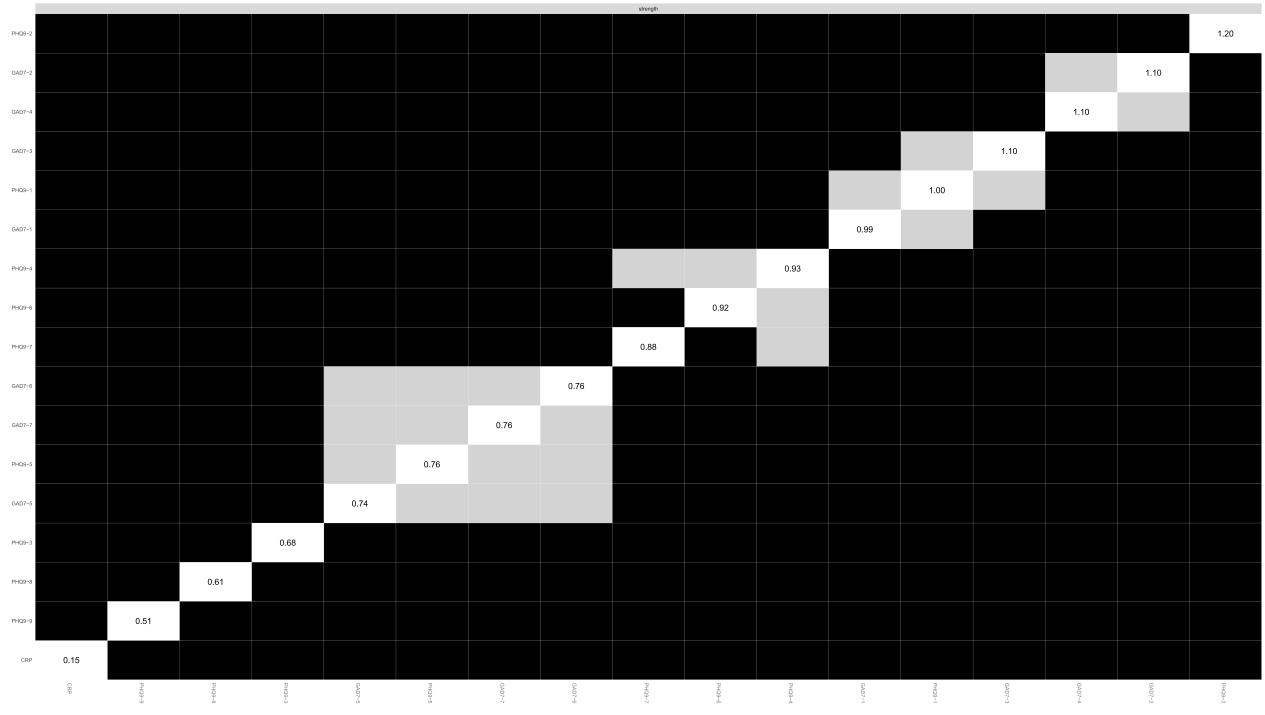


Figure S18. Estimation of node strength difference by bootstrapped difference test in never/rarely experiencing insomnia group.


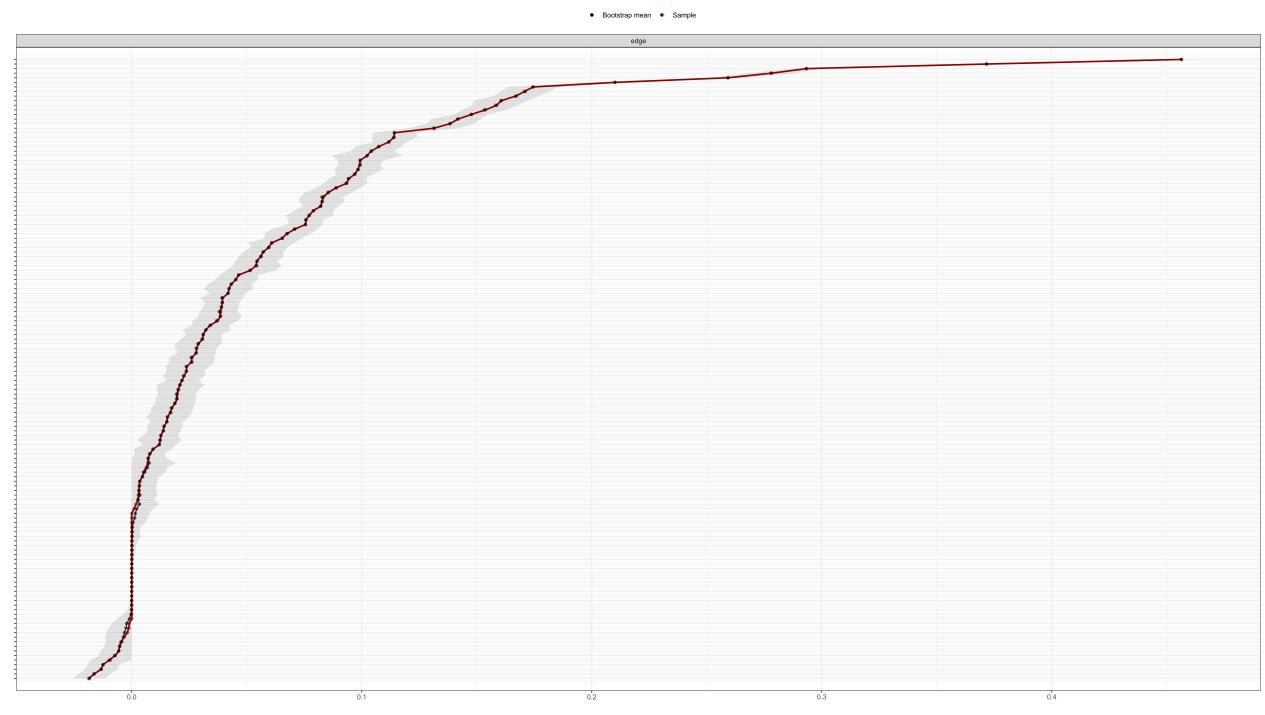


Figure S19. Bootstrapped confidence intervals of edge weights in sometimes experiencing insomnia group.


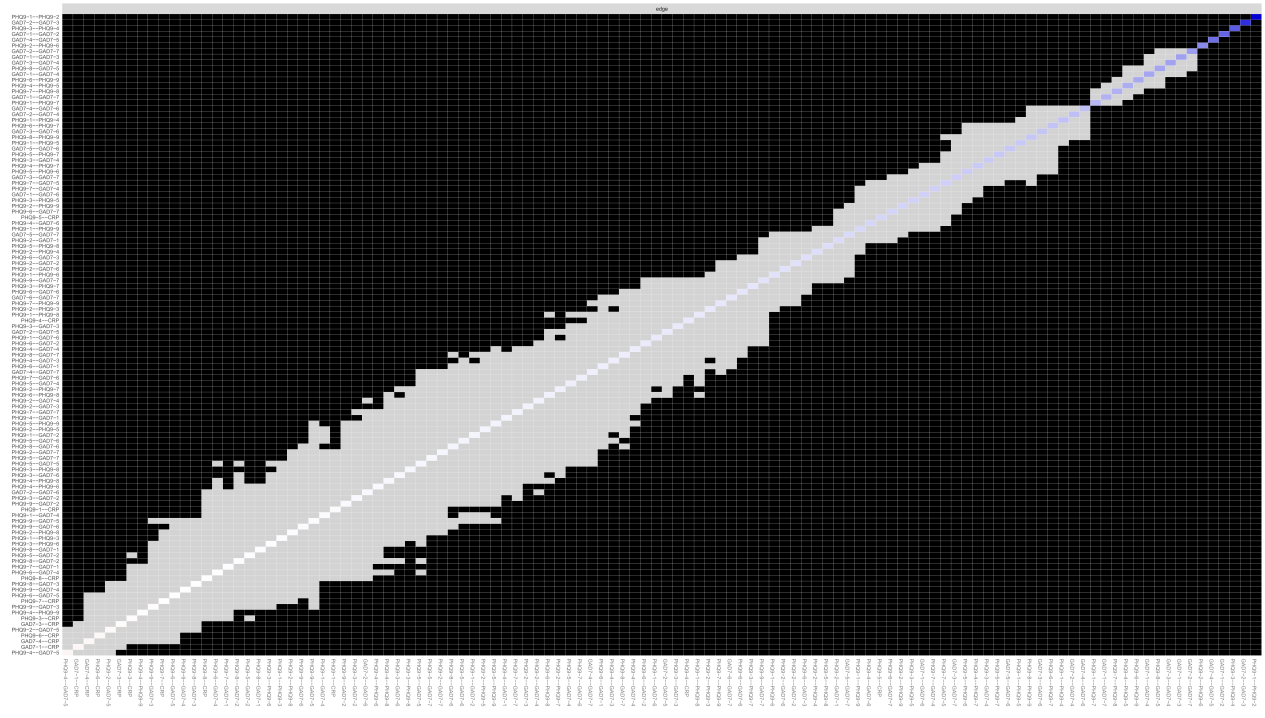


Figure S20. Estimation of edge weight difference by bootstrapped difference test in sometimes experiencing insomnia group.


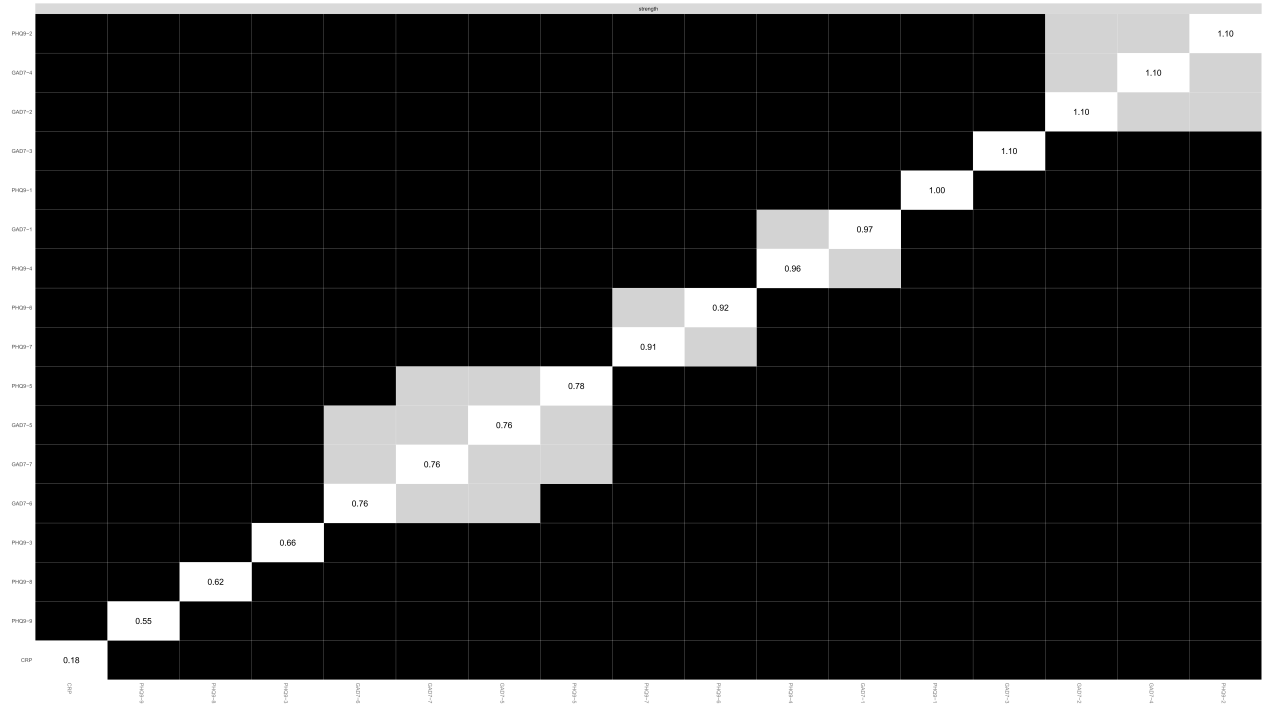


Figure S21. Estimation of node strength difference by bootstrapped difference test in sometimes experiencing insomnia group.


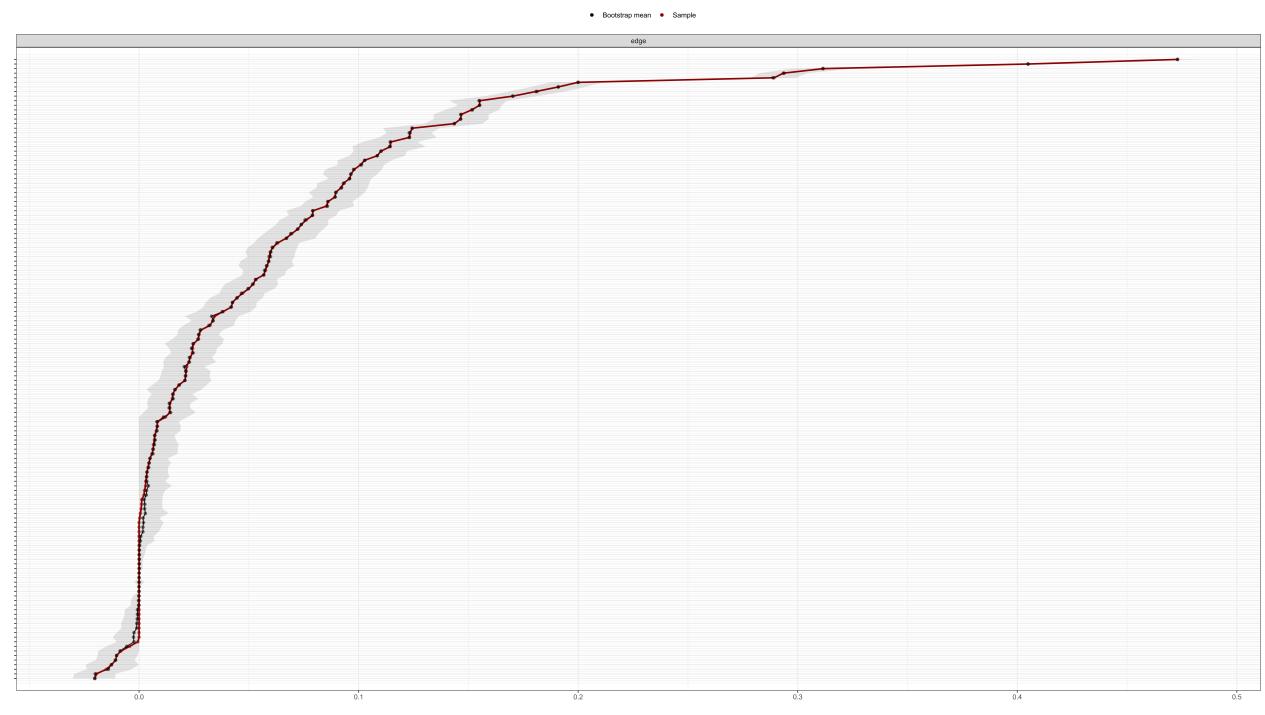


Figure S22. Bootstrapped confidence intervals of edge weights in usually experiencing insomnia group.





Figure S23. Estimation of edge weight difference by bootstrapped difference test in usually experiencing insomnia group.


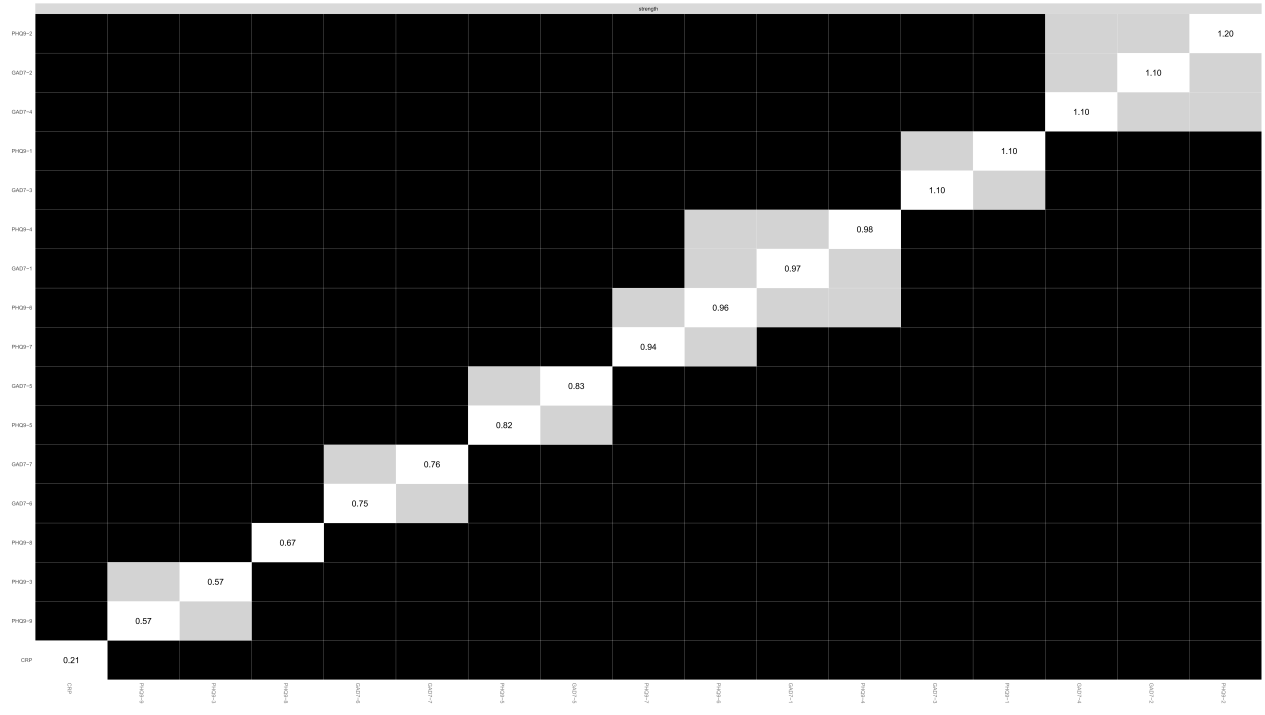


Figure S24. Estimation of node strength difference by bootstrapped difference test in usually experiencing insomnia group.


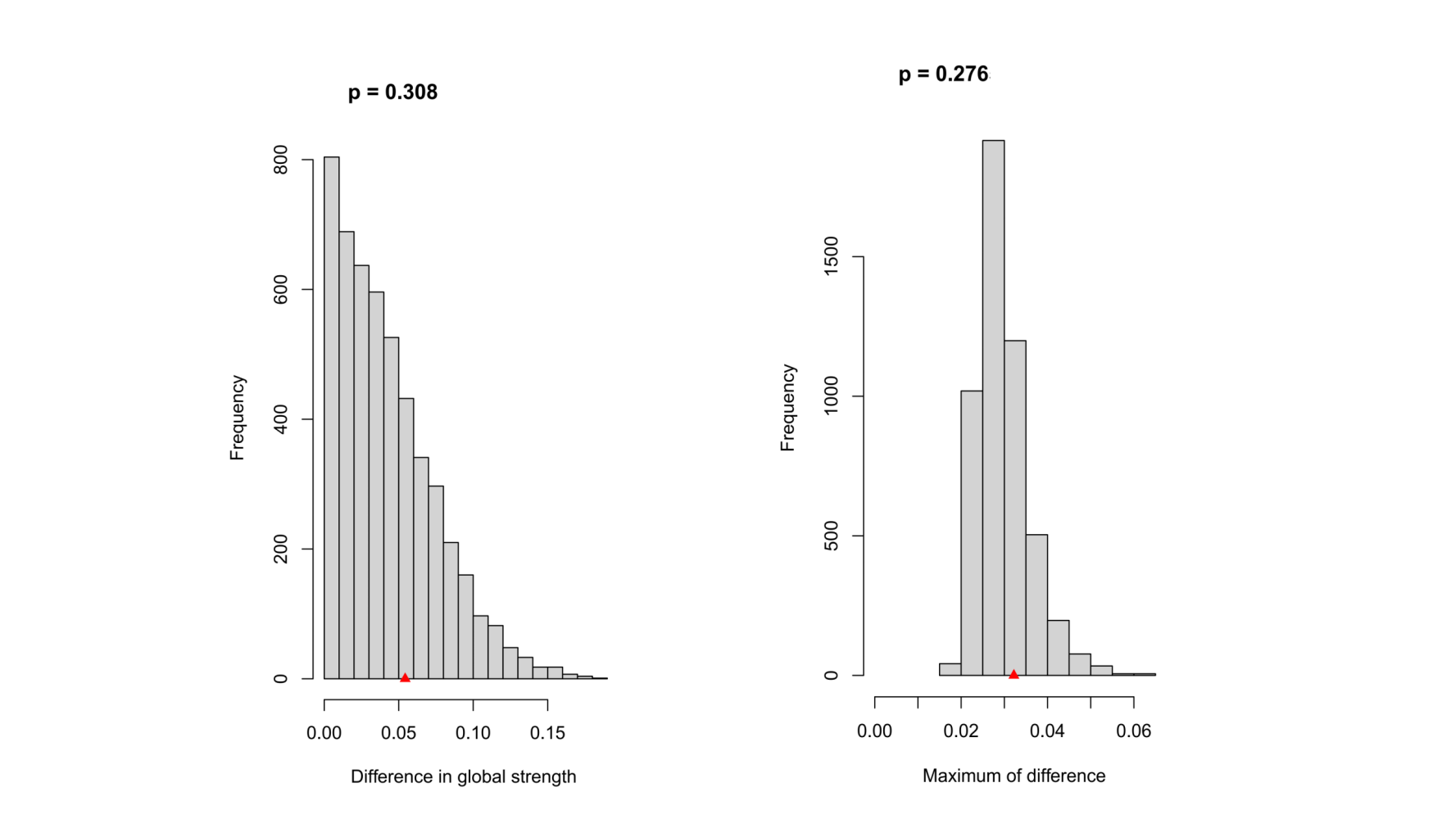


Figure S25. Comparison of network properties between never/rarely experiencing insomnia and sometimes experiencing insomnia.


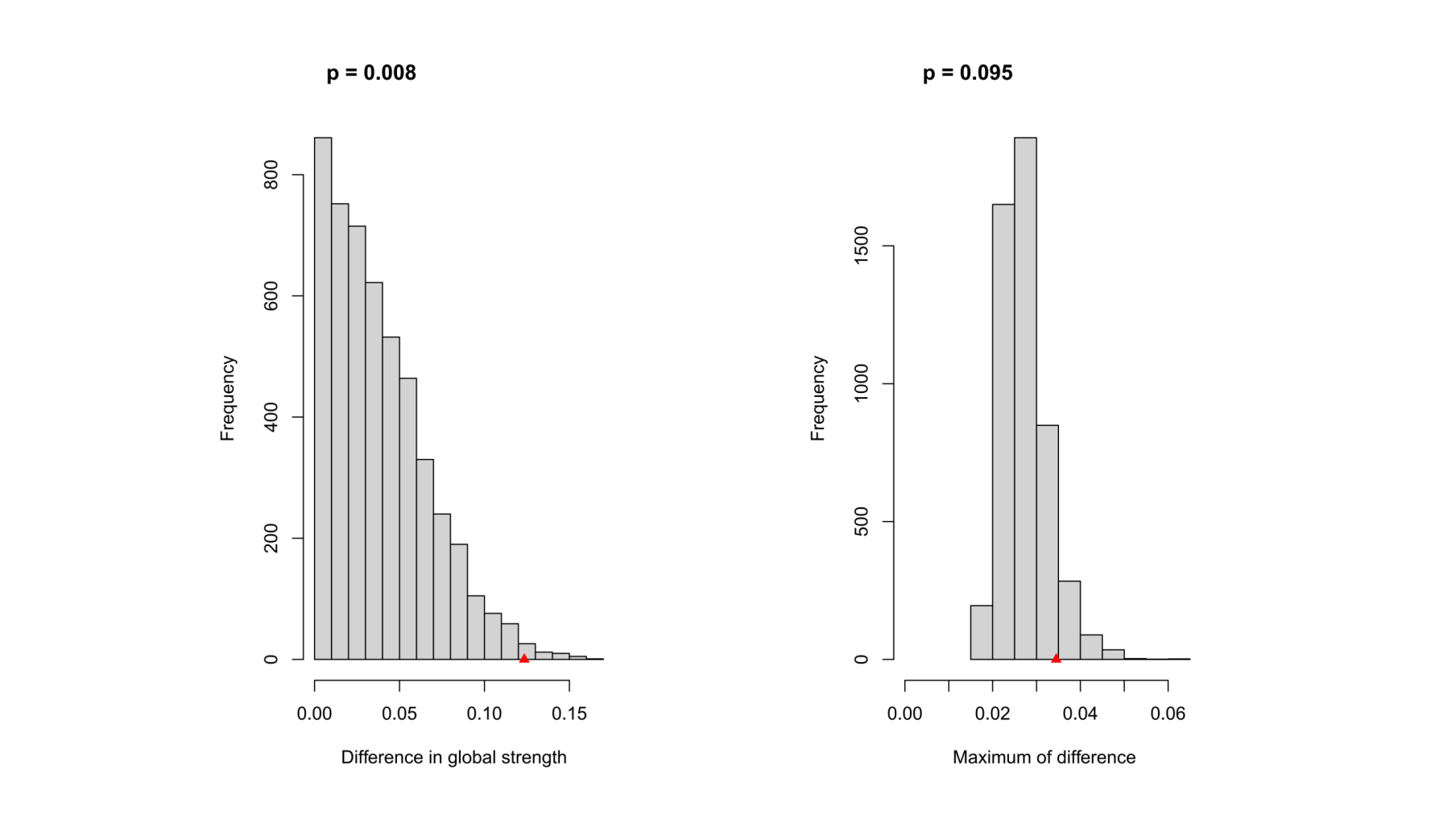


Figure S26. Comparison of network properties between sometimes experiencing insomnia and usually experiencing insomnia.
